# Inhibition of colorectal cancer progression through conformation-specific targeting of ADAM10 metalloprotease

**DOI:** 10.3389/fonc.2025.1704436

**Published:** 2026-01-27

**Authors:** Pargol Mashati, Dan Sun, Eduardo Garcia Reino, Giuseppe Militello, Nicholas Gao, Jessica A. Blandino, Nayanendu Saha, Dimitar B. Nikolov, Prem K. Premsrirut

**Affiliations:** 1 Department of Cell Biology, SUNY Downstate Medical Center, Brooklyn, NY, United States; 2 Mirimus, Inc., Brooklyn, NY, United States; 3 Structural Biology Program, Memorial Sloan-Kettering Cancer Center, New York, NY, United States

**Keywords:** ADAM10, colorectal cancer, EGFR, invasion, Notch, proliferation

## Abstract

**Introduction:**

Colorectal cancer (CRC) is the second leading cause of cancer-related deaths worldwide. Current first- and second-line therapies rely on oxaliplatin- or irinotecan-based combination chemotherapies combined with antibody-mediated inhibition of EGFR- or VEGF-dependent signaling, but these regimens are associated with significant side effects that limit long-term use and effectiveness. ADAM10 has emerged as a potential therapeutic target in CRC due to its role in activating oncogenic pathways such as Notch and EGFR; however, prior approaches targeting ADAM10 showed high toxicity. ADAM10 exists in an open, active conformation and a closed, auto-inhibited conformation, with the active form being more prevalent in tumor cells, providing a rationale for conformation-specific targeting.

**Methods:**

To investigate the therapeutic potential of 1H5, cellular viability was assessed using viability assays, and ADAM10 dependency was evaluated by shRNA-mediated knockdown. Modulation of oncogenic signaling pathways and target gene expression was validated by Western blotting and RT-qPCR, while transcriptomic changes were analyzed by bulk RNA sequencing. The effects of 1H5 on cell migration and invasion were assessed using wound-healing and transwell invasion assays, and therapeutic efficacy was evaluated in vivo using xenograft and syngeneic mouse models.

**Results:**

We show that 1H5 inhibits Notch and EGFR signaling and reduces proliferation of human CRC cell lines. We also found that 1H5 inhibits Wnt/β-catenin signaling in CRC cells and reduces their migration and invasion capacity. Finally, treatment studies in CRC cell line-derived xenograft models revealed marked antitumorigenic properties of 1H5.

**Discussion:**

Together, these findings demonstrate that selective targeting of the active conformation of ADAM10 enables simultaneous inhibition of multiple oncogenic pathways involved in CRC growth and progression and represents a promising therapeutic strategy warranting further clinical evaluation.

## Introduction

Colorectal cancer (CRC) represents the third most frequent cancer worldwide, ranking second in cancer-related mortality. It accounts for approximately 9.2% of all cancer deaths globally (1). Current treatment strategies for CRC primarily include surgical resection and cytotoxic chemotherapy plus antibody-mediated EGFR or VEGF inhibition (2). The use of chemotherapy is often associated with significant side effects. Chemotherapeutic regimens, although widely used, tend to have limited therapeutic selectivity and can cause substantial systemic toxicity, leading to adverse effects on normal tissues (3, 4). Consequently, there is a pressing need to develop novel targeted therapeutic strategies that specifically home to CRC cells to overcome these challenges. At the molecular level, CRC is driven by aberrant activation of multiple oncogenic signaling pathways, including Wnt/β-catenin, Notch, and epidermal growth factor receptor (EGFR), which collectively contribute to tumor initiation, progression, metastasis, and resistance to conventional therapies (5–7).

These oncogenic pathways are frequently initiated or amplified through proteolytic shedding events, underscoring the pivotal role of protease-mediated signaling in CRC pathogenesis (8). A key regulator of this process is the membrane-anchored metalloprotease ADAM10, which mediates the ectodomain shedding of a broad range of substrates, including the Notch receptor, EGFR ligands, and E-cadherin (8, 9). Although ADAM10 is the primary sheddase for canonical Notch activation, ADAM17 can contribute in some contexts, and several EGFR ligands are also processed by ADAM17 (10). ADAM10 functions as the sheddase for Notch, triggering extracellular cleavage followed by γ-secretase–mediated intramembrane cleavage that releases the Notch intracellular domain (NICD), which then translocates to the nucleus to regulate target gene expression (11). In addition, ADAM10 is a principal sheddase for the EGFR ligands EGF and betacellulin, whereas ADAM17 preferentially sheds ligands such as TGFα, amphiregulin, HB-EGF, and epiregulin; however, substrate usage by ADAM10 and ADAM17 can overlap and is highly context dependent (12). Ligand-bound EGFR undergoes dimerization and autophosphorylation, initiating downstream signaling cascades, including the RAS/RAF/MEK/ERK pathway, which promotes cell proliferation and survival (12). ADAM10 also cleaves adhesion molecules such as E-cadherin, disrupting cell–cell junctions and enhancing cellular detachment and motility (13–15). Through the cleavage of these substrates, ADAM10 regulates cell fate determination, promotes tumor cell proliferation, and facilitates invasion and metastasis. In addition, overexpression of ADAM10 has been correlated with aberrant signaling through Notch, ErbB family receptors, and other membrane-bound signaling molecules, contributing to a more aggressive and metastatic phenotype across multiple malignancies, including colorectal, gastric, prostate, breast, ovarian, and uterine cancers, as well as leukemia (16–19).

Given its broad impact on multiple oncogenic pathways, ADAM10 has emerged as a compelling upstream therapeutic target capable of simultaneously attenuating diverse signaling cascades. In contrast, small-molecule inhibitors aimed at downstream effectors such as Notch and EGFR have shown limited clinical success, often due to off-target effects, compensatory ligand shedding, and feedback activation (12, 20). These limitations highlight the potential advantages of targeting ADAM10 as a more effective upstream strategy. However, no highly selective ADAM10 inhibitors have advanced to human trials. To date, the only ADAM10-targeting agent assessed in clinical trials is INCB7839 (aderbasib), a dual ADAM10/17 sheddase inhibitor tested in Phase I/II trials for solid tumors and in ongoing pediatric high-grade glioma studies (NCT04295759).

Structural insights have helped clarify the autoregulatory features of ADAM10, particularly the role of its disintegrin and cysteine-rich domains in controlling its conformational state (21). More recent structural studies have further shown that ADAM10 does not function as a solitary protease but instead exists as a complex with specific tetraspanins, such as Tspan15, which regulate its maturation, trafficking, and substrate selectivity (22). This is consistent with earlier work demonstrating that ADAM10 and its partner tetraspanins are mutually dependent for stability and surface expression (23). It has been shown that ADAM10 proteolytic activity is markedly elevated in CRC compared to normal cells, providing an opportunity for tumor-specific targeting of ADAM10 (24). Recently, a conformation-specific monoclonal antibody, 1H5, was developed that selectively binds to the active, open conformation of ADAM10. This antibody targets the cysteine-rich substrate-binding domain rather than the catalytic site, enabling selective recognition of the proteolytically active form present predominantly on malignant cells. Initial studies examined its activity in a CRC cell line, suggesting a potential role in modulating Notch signaling and providing preliminary support for ADAM10 as a therapeutic target (25).

Here, in addition to evaluating the effect of 1H5 on Notch-active CRC models, we expanded our work to better understand its broader therapeutic potential across genetically diverse colorectal cancer settings. Specifically, we assessed the efficacy of 1H5 in EGFR-expressing CRC cell lines both *in vitro* and in xenograft models and further extended our investigation to immunocompetent syngeneic organoid-derived tumors. We observed that this antibody effectively suppressed the progression of various CRC cell lines in both *in vitro* and *in vivo* models, consistent with its strong inhibitory effect on ADAM10-mediated molecular pathways. These studies define the therapeutic scope of conformation-specific ADAM10 inhibition across multiple oncogenic contexts and support the advancement of 1H5 as a promising candidate for targeted CRC therapy with translational potential.

## Results

### 1H5 mAb reduces growth potential in colorectal cancer cell lines *in vitro*

**Figure 1:**
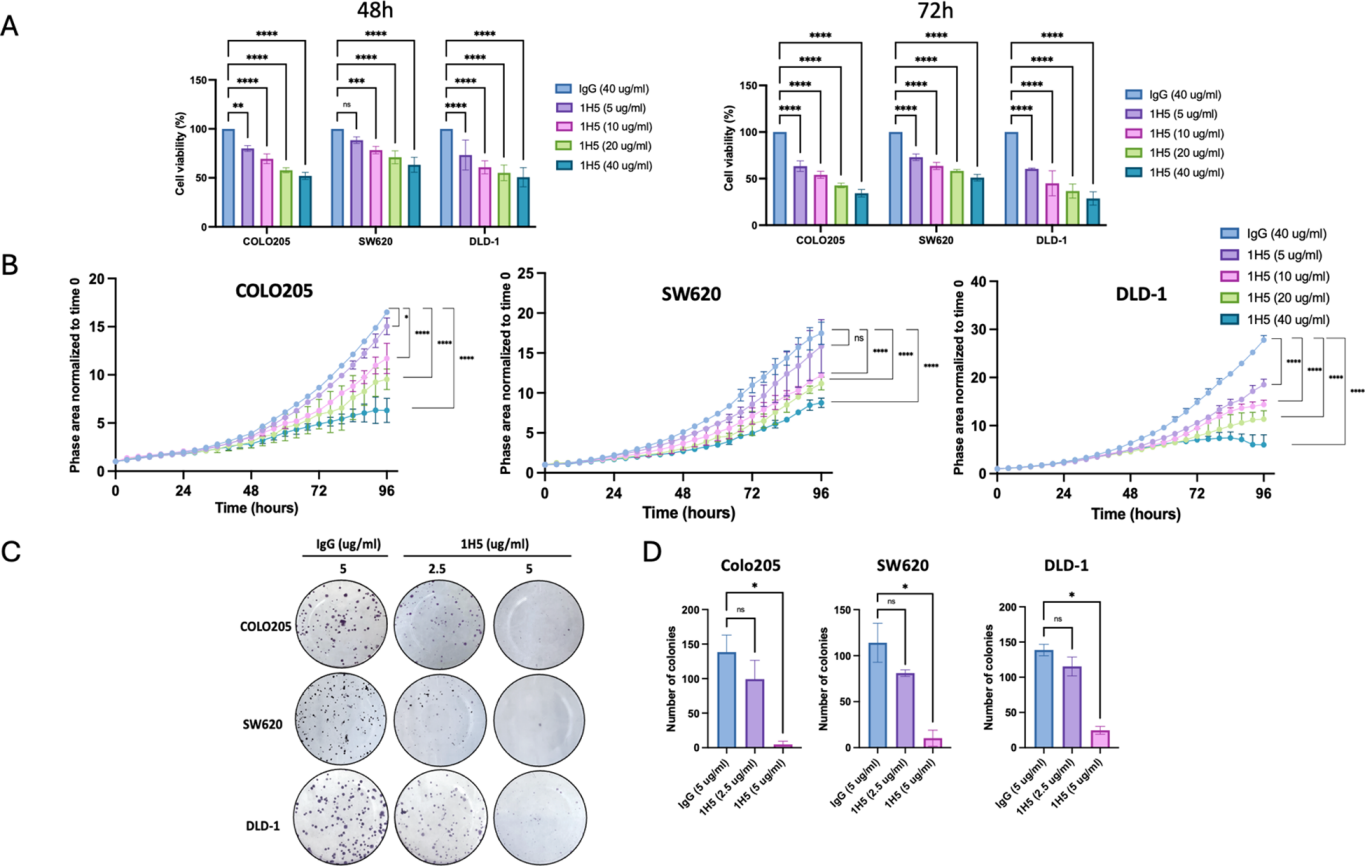
1H5 reduces viability, proliferation, and colony formation in colorectal cancer (CRC) cells. **(A)** Cell viability of COLO205, SW620, and DLD-1 cells measured by Alamar Blue after 48 h and 72 h of treatment with IgG control (40 µg/mL) or 1H5 (5–40 µg/mL). Viability values were normalized to the IgG control for visualization. For statistical testing across cell lines, normalized values were converted to Δ-viability (treatment – 100), and each time point (48 h and 72 h) was analyzed separately using two-way ANOVA (cell line × concentration) followed by Dunnett’s multiple-comparison test. **(B)** Real-time proliferation measured by IncuCyte. Phase-area confluence was tracked every 4 h and normalized to baseline. Statistical comparisons were performed using two-way ANOVA with Dunnett’s correction. **(C)** Representative images of crystal violet–stained colonies after 10 days of treatment. **(D)** Quantification of colony numbers from panel **(C)**. Group differences were analyzed using the Kruskal–Wallis test with multiple-comparison correction. Data represent mean ± SD from biological replicates. Significance levels: *p < 0.05; **p < 0.01; ***p < 0.001; ****p < 0.0001.

We first assessed the antitumorigenic efficacy of 1H5 by quantifying its impact on the viability of three genetically distinct CRC cell lines using an alamar assay measuring the metabolic activity of the cells: COLO205 (*BRAF^V600E^*), SW620 (*KRAS^G12V^*), and DLD-1 (*KRAS^G13D^*). Cells were treated with increasing concentrations of 1H5, and viability was measured after 72 h. All three cell lines showed a dose-dependent reduction in viability, with approximately 60% reduction in COLO205 and DLD-1 and ~40% reduction in SW620 (**Figure 1A**). Importantly, in normal fetal human colon (FHC) cells, 1H5 treatment resulted in modest changes in Alamar Blue signal that did not reach statistical significance and lacked the dose-dependent suppression observed in colorectal cancer cell lines (**Supplementary Figure 1**), supporting a preferential effect of 1H5 on cancer cells.

To further assess the impact on tumor cell growth, we performed live-cell imaging and observed a marked reduction in proliferation across all three lines, with the strongest effects again seen in COLO205 and DLD-1 (**Figure 1B**). Long-term clonogenic potential was also assessed via colony formation assays. Treatment with 5 μg/mL of 1H5 led to a significant, dose-dependent decrease in colony formation across all CRC lines tested (**Figures 1C, D**). Together, these results demonstrate that 1H5 effectively suppresses CRC cell metabolic activity, proliferation, and clonogenic growth *in vitro*, with enhanced sensitivity in COLO205 and DLD-1 cells.

### Inducible ADAM10 knockdown confirms target specificity of 1H5 in CRC cell lines

To determine the target specificity of 1H5, we next aimed to selectively deplete ADAM10 expression and subsequently measure 1H5 activity. Initial attempts to generate ADAM10 knockout CRC cell lines using CRISPR gene editing were unsuccessful, likely due to decreased proliferative potential of ADAM10-depleted cells (data not shown). Therefore, we resorted to the use of an all-in-one inducible RNA interference (RNAi) lentiviral expression cassette (26), whereby ADAM10 knockdown could be induced by doxycycline (dox) administration. CRC cells were transduced with a GFP-linked shRNA targeting ADAM10 at either nucleotide position 189 (shADAM10_1) or 2321 (shADAM10_2). Following dox induction, over 70% of cells expressed GFP, indicating efficient transduction (**Supplementary Figure 2A**), with substantial reduction of ADAM10 protein levels after dox treatment in all three cell lines, whereas control cells expressing a non-targeting shRNA (shRenilla) maintained robust ADAM10 expression (**Supplementary Figure 2A**).

**Figure 2:**
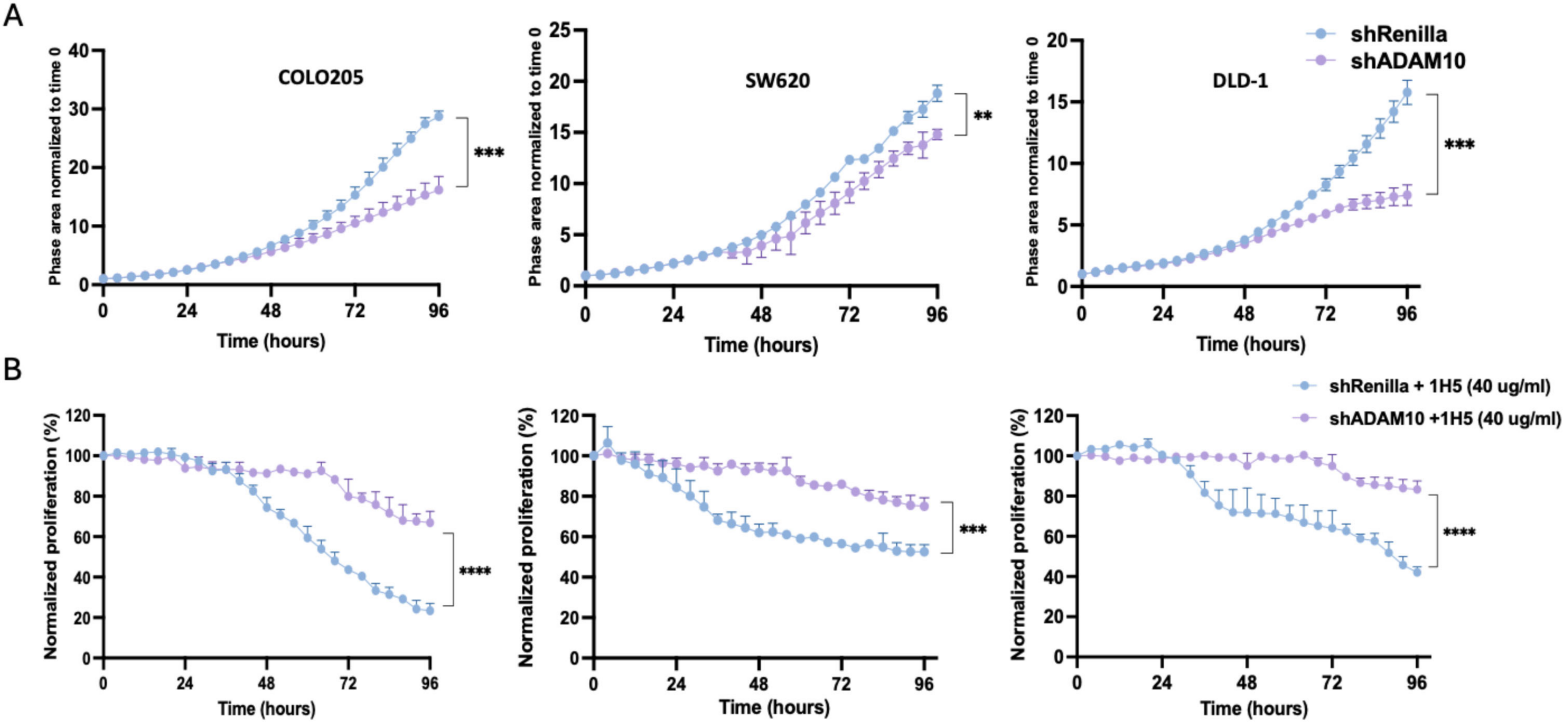
1H5 specifically targets ADAM10. **(A)** Proliferation curves of shRenilla and shADAM10 knockdown cells (COLO205, SW620, and DLD-1) measured by live-cell imaging over 96 h following doxycycline induction. Cells were seeded in the presence of 1 µg/mL doxycycline to induce shRNA expression. Phase area was normalized to baseline (0 h). **(B)** Relative proliferation of doxycycline-induced shRenilla and shADAM10 cells following treatment with 40 µg/mL 1H5. 1H5 was added 24 h after seeding, and proliferation was monitored by live-cell imaging over time. Data represent three biological replicates and are shown as mean ± SD. ****p < 0.0001, ***p < 0.001, **p < 0.01 by two-way ANOVA.

shADAM10_1 demonstrated more robust knockdown efficiency and was therefore used for subsequent experiments. Proliferation assays using live-cell imaging revealed that ADAM10-depleted cells exhibited slower proliferation compared to control shRenilla cells, particularly in COLO205 and DLD-1 (**Figure 2A**). To assess whether the antiproliferative effect of 1H5 is mediated through ADAM10, both control and shADAM10_1 cells were treated with 40 μg/mL of 1H5. While control cells showed significant growth inhibition, ADAM10 knockdown cells displayed marked resistance to treatment (**Figure 2B**). These findings confirm that 1H5 exerts its antiproliferative activity through ADAM10, supporting its target specificity and validating ADAM10 as a functional dependency in CRC.

### 1H5 inhibits Notch signaling in colorectal cancer cell lines

**Figure 3:**
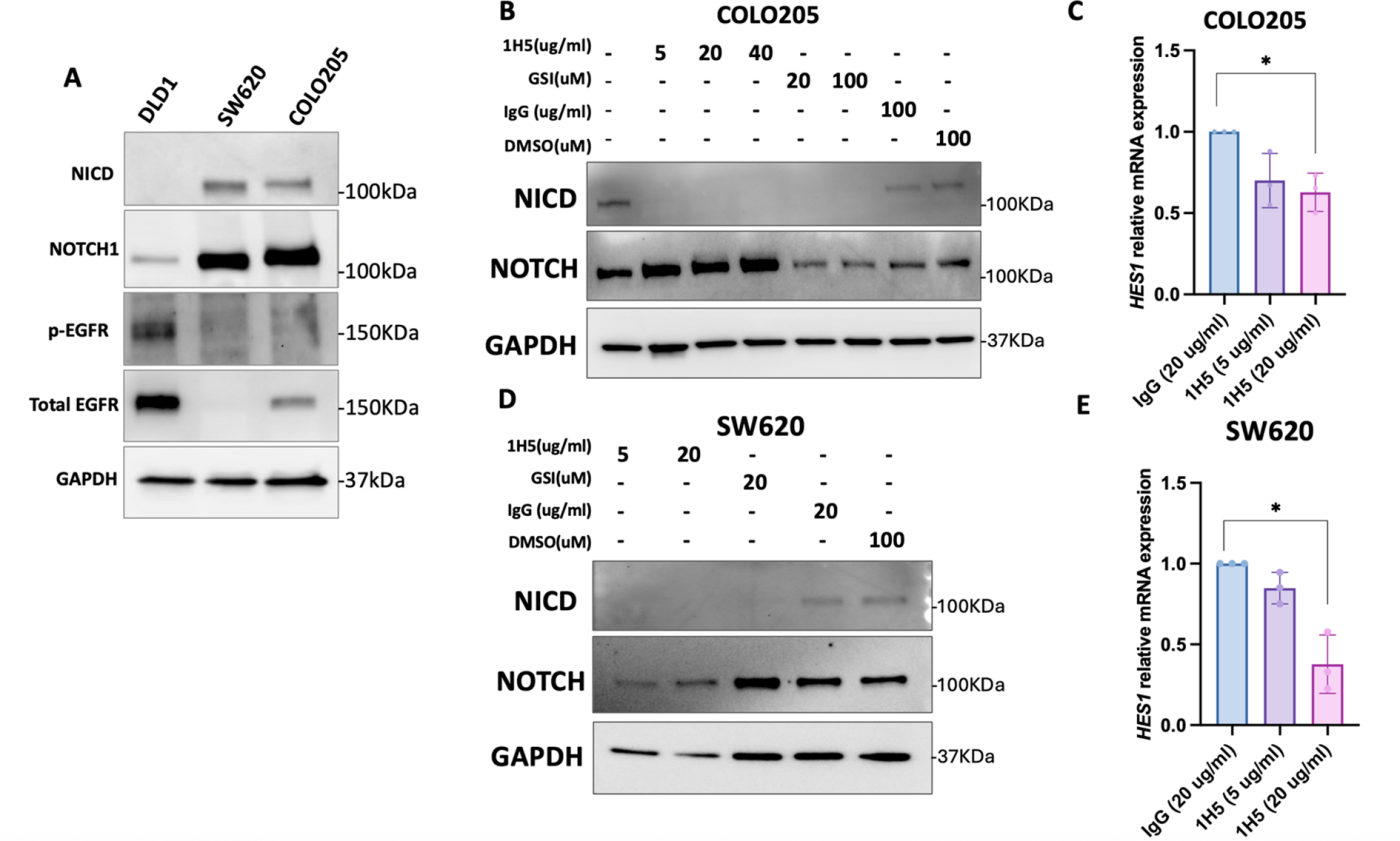
1H5 inhibits Notch signaling in CRC cell lines. **(A)** Basal protein expression of NICD, NOTCH1, phosphorylated EGFR (p-EGFR), and total EGFR in DLD-1, SW620, and COLO205 cells. GAPDH served as a loading control. Western blots were performed using two biological replicates. **(B)** Western blot analysis of NICD and NOTCH1 in COLO205 cells treated with 1H5 (5–40 µg/mL), γ-secretase inhibitor (GSI; 20 or 100 µM), IgG control (100 µg/mL), or DMSO (100 µM) for 48 **(h)** GAPDH was used as a loading control. Two biological replicates were performed. **(C)** HES1 mRNA expression in COLO205 cells treated with IgG control (20 µg/mL) or 1H5 (5 or 20 µg/mL) for 48 h Data are presented as mean ± SD. Statistical significance was assessed using the Kruskal–Wallis test followed by Dunn’s multiple-comparison test; p < 0.05. **(D)** Western blot analysis of NICD and NOTCH1 in SW620 cells treated with 1H5 (5 or 20 µg/mL), GSI (20 µM), IgG control (20 µg/mL), or DMSO (100 µM) for 48 (h) GAPDH was used as a loading control. Two biological replicates were performed. **(E)** HES1 mRNA expression in SW620 cells treated with IgG control (20 µg/mL) or 1H5 (5 or 20 µg/mL) for 48 (h) Data are presented as mean ± SD. Statistical significance was assessed using the Kruskal–Wallis test with Dunn’s *post hoc* test; *p < 0.05.

We next characterized downstream targets of ADAM10, focusing on Notch and EGFR signaling in our CRC cell lines. Immunoblot analyses revealed that COLO205 and SW620 cells exhibited active Notch signaling, while DLD-1 cells predominantly displayed activation of the EGFR pathway (**Figure 3A**). Treatment of COLO205 and SW620 cells with increasing concentrations of 1H5 demonstrated inhibition of Notch signaling, as evidenced by loss of NICD expression despite continued total Notch expression. Notable suppression was observed at concentrations as low as 5 μg/mL, comparable to the effects of γ-secretase inhibitor (GSI), which blocks NICD release through γ-secretase inhibition (**Figures 3B, D**). Consistent with decreased NICD levels, quantitative PCR analysis showed reduced *HES1* mRNA expression following 1H5 treatment in both cell lines, confirming effective Notch pathway inhibition (**Figures 3C, E**). We further investigated the efficacy of 1H5 in a dose-response experiment using COLO205 xenograft models. Mice were treated with either 10 mg/kg or 20 mg/kg of 1H5, with both concentrations resulting in significant inhibition of tumor growth after 21 days of treatment. Notably, this antitumor effect was achieved without any observable changes in body weight, indicating good tolerability of the treatment. Assessment of Notch signaling in harvested tumors revealed a significant reduction in Notch activity in 1H5-treated animals, further confirming effective pathway inhibition *in vivo* (**Supplementary Figures 3A–D**).

### 1H5 inhibits EGFR/ERK signaling and suppresses metabolic gene expression in DLD-1 cells

**Figure 4:**
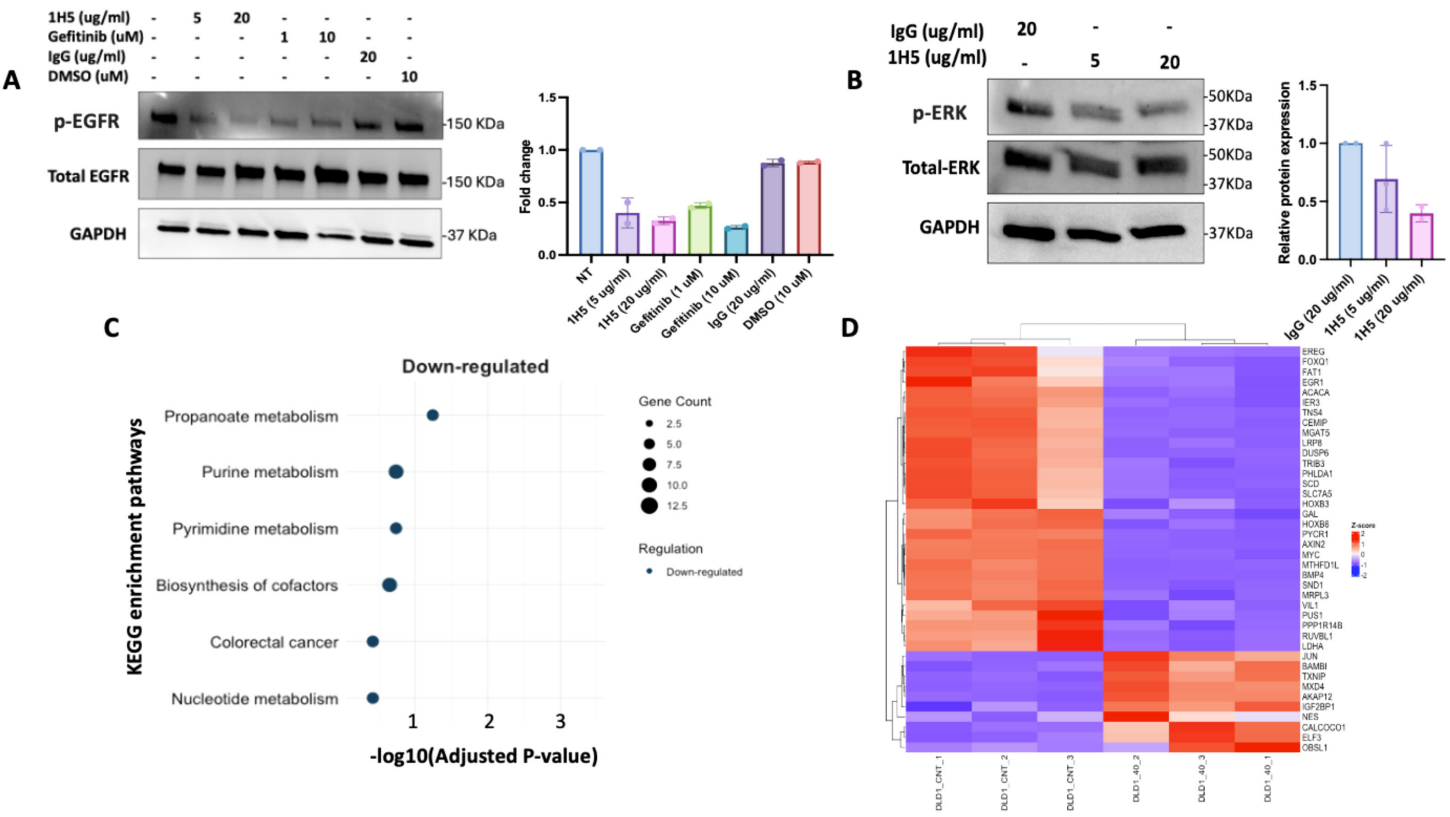
1H5 inhibits EGFR signaling and downregulates metabolic and oncogenic pathways. **(A)** Western blot analysis of phosphorylated EGFR (p-EGFR) and total EGFR in DLD-1 cells treated with 1H5 (5–20 µg/mL), IgG control (20 µg/mL), gefitinib (1–10 µM), or DMSO (10 µM) for 48 (h) GAPDH served as a loading control. p-EGFR and total EGFR blots were performed twice. **(B)** Western blot analysis of phosphorylated ERK (p-ERK) and total ERK in DLD-1 cells treated with 1H5 (5–20 µg/mL) or IgG control (20 µg/mL) for 48 (h) GAPDH served as a loading control. p-ERK and total ERK blots were performed twice. **(C)** KEGG pathway enrichment analysis of downregulated genes in DLD-1 cells treated with 40 µg/mL 1H5 for 48 h compared with IgG control (40 µg/mL), indicating significant enrichment of metabolic and cancer-related pathways. **(D)** Heatmap of differentially expressed genes in DLD-1 cells treated with 20 µg/mL 1H5 for 48 h compared with IgG control.

Given that DLD-1 colorectal cancer cells display active EGFR signaling in the absence of Notch activation, we hypothesized that 1H5 may inhibit this alternative oncogenic pathway by blocking ADAM10-mediated ligand shedding upstream of EGFR activation. To test this, DLD-1 cells were treated with 5 or 20 μg/mL of 1H5. Immunoblotting revealed a dose-dependent reduction in phosphorylated EGFR, comparable to the effect of gefitinib, a clinically approved EGFR tyrosine kinase inhibitor (**Figure 4A**). Total EGFR levels remained unchanged, indicating that 1H5 specifically impairs receptor activation rather than protein expression. We next examined downstream MAPK signaling by assessing ERK phosphorylation. 1H5 treatment led to a corresponding decrease in phosphorylated ERK (p-ERK) levels, further supporting suppression of EGFR-driven signaling (**Figure 4B**).

To evaluate the broader transcriptomic impact of 1H5, we performed RNA sequencing of 1H5-treated DLD-1 cells versus control IgG-treated cells. KEGG pathway enrichment analysis revealed significant downregulation of multiple metabolic pathways, including purine and pyrimidine metabolism (**Figure 4C**). Consistent with this, we observed reduced expression of several key metabolic regulators such as MYC, LDHA, PYCR1, MGAT5, CXCL5, SCD, LRP8, EGR1, and SLC7A5 (**Figure 4D**), which was further validated by qRT-PCR (**Supplementary Figures 4A, B**). In contrast, a subset of stress- and injury-responsive genes was upregulated, including JUN, a MAPK/JNK-regulated transcription factor whose induction likely reflects an adaptive stress or inflammatory response to ADAM10 inhibition.

Together, these findings demonstrate that 1H5 effectively inhibits EGFR/ERK signaling and, in addition, disrupts the expression of metabolic and biosynthetic genes essential for CRC proliferation, highlighting its therapeutic potential even in CRC cells lacking active Notch signaling.

### 1H5 effectively inhibits tumor growth *in vivo* in DLD-1 xenograft mouse models

**Figure 5:**
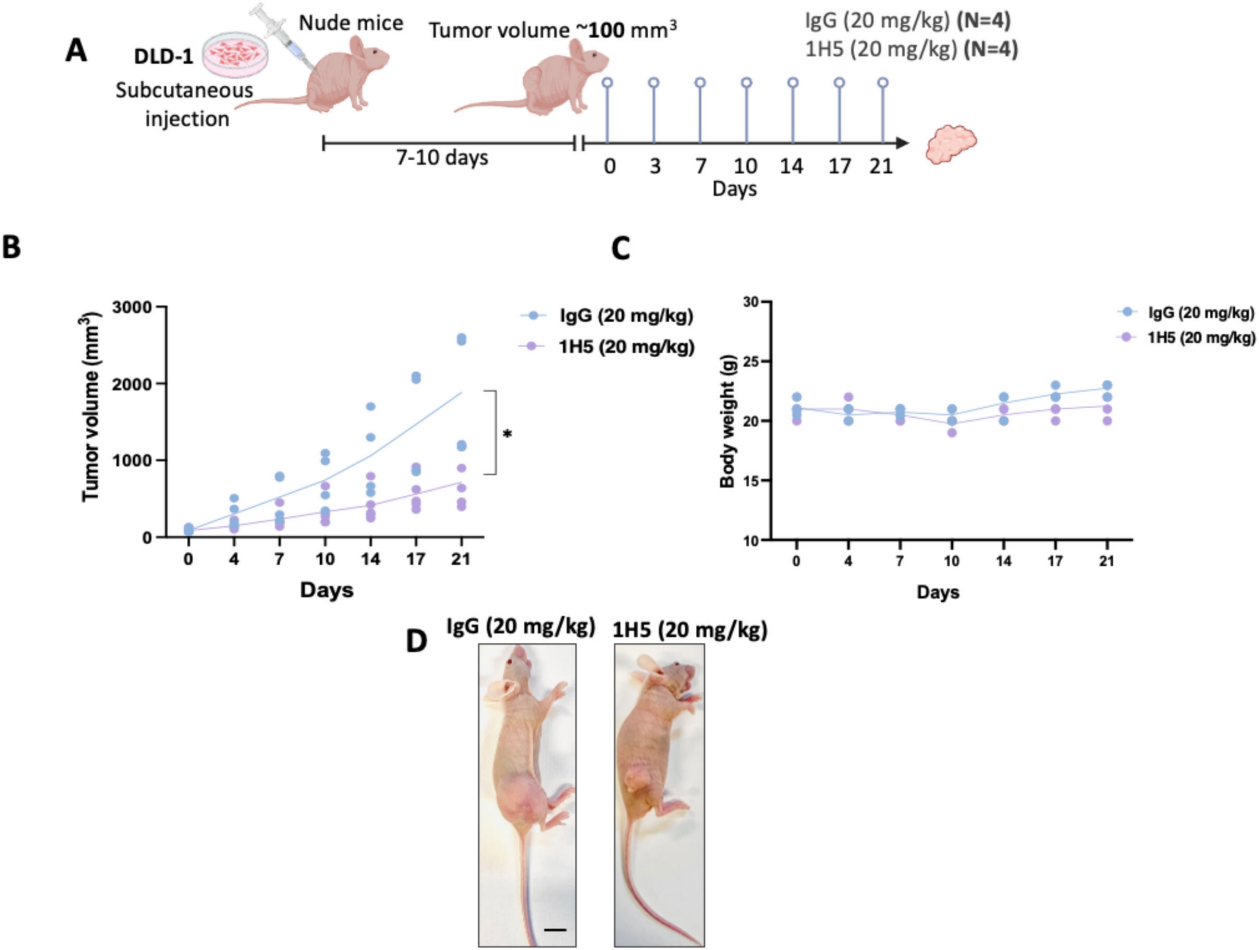
1H5 effectively inhibits tumor growth *in vivo* in DLD-1 xenograft mouse models. **(A)** Schematic of the *in vivo* study design: nude mice bearing subcutaneous DLD-1 tumors were treated twice weekly with 1H5 (20 mg/kg) or IgG control for 21 days (n = 4 per group). **(B)** Tumor volume progression over the treatment period. Statistical significance at the final endpoint (day 21) was assessed using the Mann–Whitney test; *p < 0.05. **(C)** Body weight monitoring throughout the study showed no evidence of treatment-related toxicity. **(D)** Representative images of mice at day 21 illustrate the visibly reduced tumor size in the 1H5-treated group compared with IgG controls.

Given the potent *in vitro* activity of 1H5 in DLD-1 cells, we next sought to evaluate its therapeutic efficacy *in vivo*. A DLD-1 xenograft model was established by subcutaneous engraftment of cells into female NU/J nude mice (Foxn1^nu/nu^), followed by twice weekly administration of either 20 mg/kg of 1H5 or isotype IgG control for 21 days (**Figure 5A**). Treatment with 1H5 led to a significant suppression of tumor growth compared to control-treated animals, as reflected by tumor volume measurements over time. Importantly, no significant weight loss was observed, indicating that 1H5 was well tolerated (**Figures 5B–D**). These findings demonstrate the *in vivo* antitumor efficacy and tolerability of 1H5, supporting its continued development as a therapeutic candidate for EGFR-active colorectal cancer.

### 1H5 reduces migration and invasion and modulates Wnt/β-catenin signaling in SW620 metastatic colorectal cancer cells

**Figure 6:**
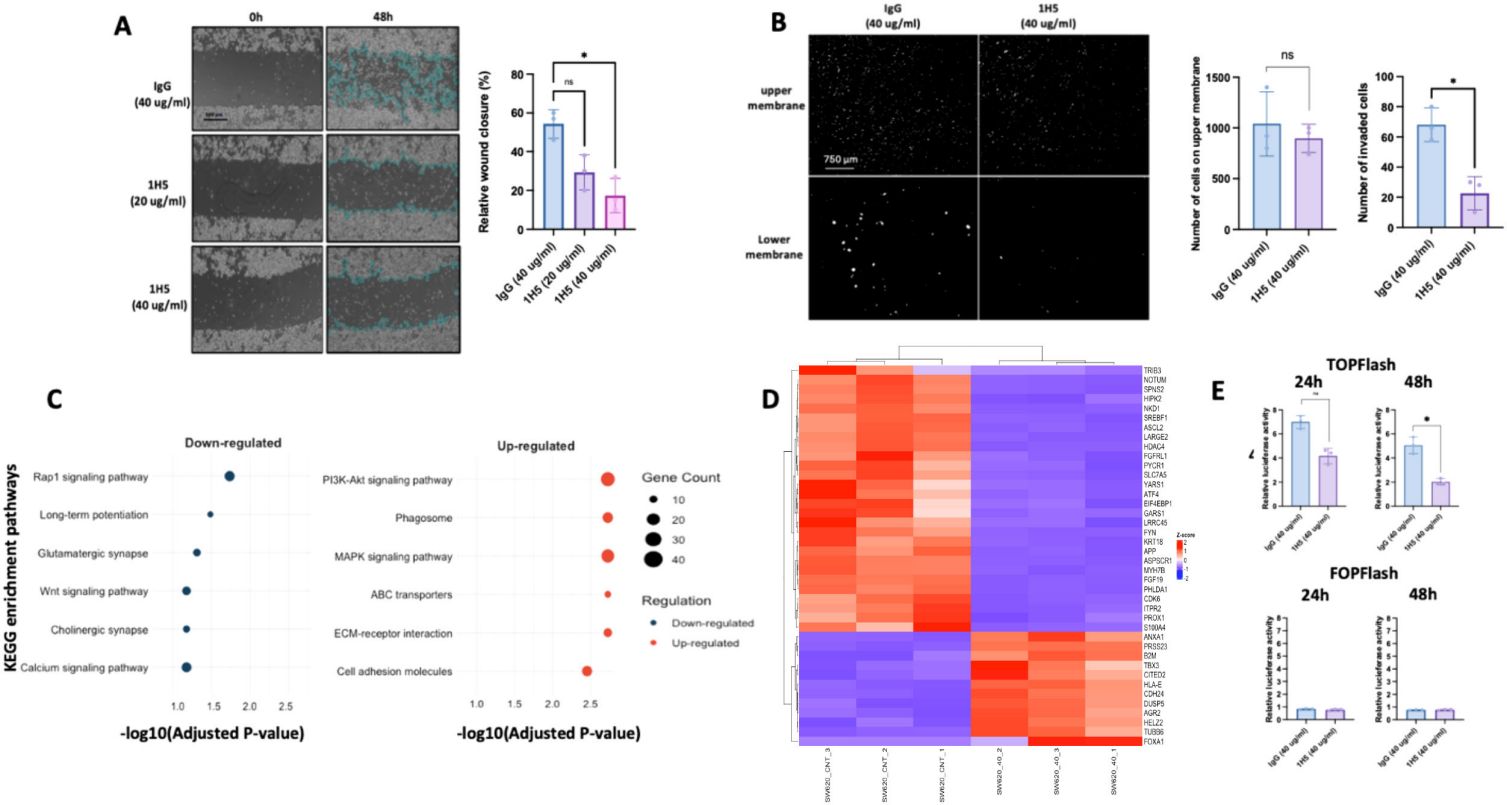
1H5 reduces migration and invasion in SW620 cells and suppresses Wnt/β-catenin signaling. **(A)** Scratch wound healing assay of SW620 cells treated with IgG control (40 µg/mL) or 1H5 (20 or 40 µg/mL). Representative images at 0 and 48 h are shown, with quantification of relative wound closure on the right. Data represent biological replicates, shown as mean ± SD, and were analyzed using the Kruskal–Wallis test with Dunn’s *post hoc* test. **(B)** Transwell invasion assay of SW620 cells treated with IgG control (40 µg/mL) or 1H5 (40 µg/mL). Representative images of the upper and lower membranes are shown, with quantification of invaded cells on the right. Data represent biological replicates, shown as mean ± SD, and were analyzed using the Mann–Whitney test. **(C)** KEGG pathway enrichment analysis of significantly downregulated (blue) and upregulated (red) pathways in SW620 cells treated with 1H5 (40 µg/mL) for 48 h compared with IgG control. **(D)** Heatmap of differentially expressed genes in SW620 cells treated with 1H5 (40 µg/mL) for 48 h compared with IgG control. **(E)** TOPFlash and FOPFlash luciferase reporter assays in SW620 cells treated with IgG control (40 µg/mL) or 1H5 (40 µg/mL) for 24 and 48 (h) TOPFlash contains wild-type TCF/LEF binding sites and reports β-catenin–TCF transcriptional activity, whereas FOPFlash contains mutated TCF/LEF sites and serves as a negative control. Data represent biological replicates, shown as mean ± SD, and were analyzed using the Mann–Whitney test.

Given that metastasis is a major cause of mortality in CRC patients, we assessed the effect of 1H5 on the migratory and invasive properties of the metastatic SW620 cell line. Scratch wound assays demonstrated that treatment with 1H5 (20 or 40 μg/mL) significantly reduced cell migration after 48 h compared to IgG-treated controls (**Figure 6A**). Consistent with these findings, transwell invasion assays revealed a marked reduction in the number of cells invading through the lower membrane following 1H5 treatment (**Figure 6B**). To explore the molecular mechanisms underlying these phenotypic changes, RNA sequencing was performed on SW620 cells treated with 1H5. Pathway enrichment analysis revealed significant downregulation of the Wnt/β-catenin signaling pathway (**Figure 6C**). Transcriptomic analysis showed significant downregulation of key Wnt/β-catenin target genes, including *NKD1*, *NOTUM*, and *S100A5*, which ranked among the top 40 differentially expressed genes. Notably, there was also marked upregulation of *DKK1*, a well-established Wnt antagonist (**Figure 6D**, **Supplementary Figure 5A**). To validate these findings, we performed RT-qPCR, which confirmed the differential expression of these Wnt-related genes following 1H5 treatment (**Supplementary Figure 5A**). To functionally evaluate the impact on Wnt/β-catenin signaling, we conducted a TOPFlash reporter assay, which quantifies β-catenin/TCF-mediated transcriptional activity via luciferase expression driven by TCF/LEF response elements. Cells treated with 1H5 displayed significantly reduced β-catenin-driven luciferase activity compared to IgG-treated controls (**Figure 6E**), confirming effective inhibition of Wnt signaling upon ADAM10 blockade (**Figure 6D**). In addition to these transcriptomic changes, Western blot analysis demonstrated that 1H5 treatment enhanced E-cadherin expression, supporting increased cell adhesion, and promoted cytoplasmic accumulation of β-catenin, while nuclear β-catenin levels remained largely unchanged—an observation that warrants further investigation (**Supplementary Figure 5C**).

### 1H5 attenuates tumor growth in KAP colorectal cancer organoids *in vitro* and in syngeneic mouse models

**Figure 7:**
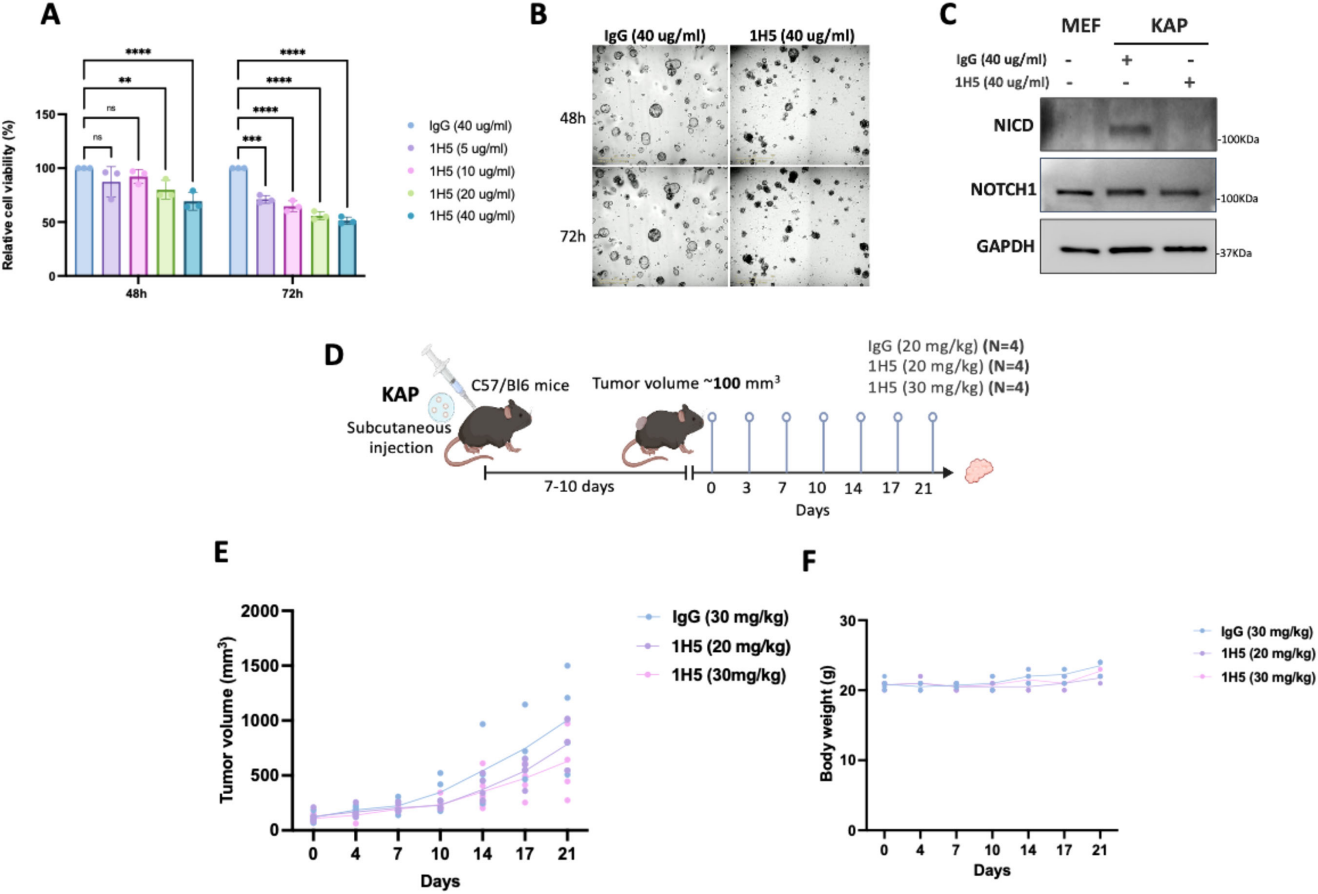
1H5 inhibits tumor growth in KAP colorectal cancer organoids *in vitro* and in syngeneic mouse models. **(A)** Cell viability assay of KAP cells treated with IgG control (40 µg/mL) or increasing concentrations of 1H5 (5–40 µg/mL) for 48 and 72 (h) Data are shown as mean ± SD and analyzed using two-way ANOVA with Dunnett’s multiple-comparison test. **(B)** Brightfield images of 3D KAP spheroids treated with IgG control (40 µg/mL) or 1H5 (40 µg/mL) for 48 and 72 (h) **(C)** Western blot analysis of NICD and NOTCH1 in KAP cells compared with MEFs following treatment with 1H5 (40 µg/mL). GAPDH served as a loading control. **(D)** Schematic of the syngeneic study design: C57BL/6 mice bearing subcutaneous KAP tumors (~100 mm³) were treated twice weekly with IgG control (30 mg/kg) or 1H5 (20 or 30 mg/kg) for 21 days. **(E)** Final tumor volumes at day 21 were analyzed using the Kruskal–Wallis test with Dunn’s *post hoc* comparisons. **(F)** Body weight monitoring throughout the treatment period. Data represent mean ± SD from biological replicates. Signi cance levels: *p < 0.05; **p < 0.01; ***p < 0.001; ****p < 0.0001.

It is well established that immune cells significantly shape the tumor microenvironment of colorectal carcinomas and that immune cell infiltration can have both positive and negative effects on the success of systemic therapies, depending on the context (27). Therefore, we set out to evaluate the efficacy of 1H5 in a more physiologically relevant, immunocompetent setting, namely genetically engineered 3D mouse colorectal cancer organoids. KAP organoids (*Kras^G12D^;Apc^+/−^;Trp53^-/-^*) and BAP organoids (*Braf^V600E^; Apc-/-; Trp53+/-*) harboring common CRC driver mutations and exhibiting activated Notch signaling were utilized. Treatment with 1H5 significantly reduced organoid viability at both 48 and 72 h in each organoid model (**Figures 7A, B**; **Supplementary Figure 6A**). Consistent with the proposed mechanism of action, immunoblot analysis confirmed inhibition of Notch pathway activation following treatment (**Figure 7C**). To assess therapeutic potential *in vivo*, KAP organoids were subcutaneously implanted into the flanks of immunocompetent C57BL/6 WT mice. In parallel, BAP organoids were implanted using the same protocol but failed to establish tumors. Biweekly administration of 1H5 at 30 mg/kg resulted in a significant reduction in tumor growth compared to IgG-treated controls (**Figures 7D–F**). Treatment with 20 mg/kg also reduced tumor growth, but to a lesser extent. To rule out the development of a mouse anti-human antibody (MAHA) response against the therapeutic antibody, serum samples were collected after each injection. MAHA assays performed on these samples showed no detectable antibody response throughout the treatment course (**Supplementary Figure 6**).

## Discussion

ADAM10 is a key sheddase that regulates the proteolytic release of multiple transmembrane proteins, including growth factor ligands and adhesion molecules. In CRC, ADAM10 is an essential factor that promotes elevated activity of downstream proteins contributing to enhanced proliferative, invasive, and survival signaling (16, 17). Despite its central role in cancer progression, targeting ADAM10 has been challenging due to its structural similarity to other ADAM proteases, which limits small-molecule specificity. Here, we evaluate 1H5, a conformation-specific monoclonal antibody that selectively binds the open, active form of ADAM10, which is predominantly expressed on malignant cells. While previous studies on ADAM10 in colorectal cancer have largely been limited to its role in Notch signaling (18, 24, 25, 28), our work offers broader mechanistic insight into its functional contribution to CRC progression and the therapeutic potential of targeting its active conformation across genetically diverse CRC models.

Our data establish that 1H5 exerts broad antitumor activity across heterogeneous CRC models. Beyond suppressing Notch signaling in CRC cell lines with high Notch activation—resulting in marked tumor growth attenuation at relatively low doses in xenograft models—1H5 also demonstrated potent activity in an EGFR-driven CRC context. ADAM10 is known to shed EGFR ligands such as EGF and betacellulin, and in our EGFR-driven CRC models (12), 1H5 suppressed EGFR and ERK activation in a manner consistent with reduced ligand-driven signaling, although direct measurement of ligand shedding was not performed. These effects extended *in vivo*, where 1H5 treatment produced robust tumor growth suppression with no apparent signs of toxicity, as assessed by body-weight measurements and general animal condition.

These findings in EGFR-active cell lines are particularly significant. First, ligand cleavage has been shown to be a driver of resistance in ERBB- and EGFR-dependent pathways, suggesting that inhibition of ligand shedding represents a distinct therapeutic vulnerability that may help overcome resistance in CRC (29). However, it is important to recognize that ADAM10 is not the only sheddase regulating ERBB ligands; ADAM17 is also a major contributor to EGFR ligand activation and can compensate for loss of ADAM10 activity in certain contexts (30). Thus, the impact of 1H5 may be influenced by the relative expression or activity of ADAM17 in different CRC models, and future studies will be needed to determine whether ADAM17-mediated shedding modulates or limits the full therapeutic effect of ADAM10 blockade. Second, another established mechanism of resistance to EGFR inhibitors is compensatory activation of other ERBB family members, such as HER3 in colorectal cancer (31). Given ADAM10’s role in mediating the cleavage of multiple ERBB ligands (32, 33), inhibition by 1H5 has the potential to simultaneously suppress these compensatory pathways, thereby broadening its therapeutic benefit, particularly in combination with EGFR-targeting antibodies such as cetuximab or panitumumab. Importantly, its selectivity for the active conformation of ADAM10 ensures precise tumor targeting with minimal impact on normal tissues.

We further demonstrated that the ability of 1H5 to attenuate Wnt/β-catenin signaling in metastatic CRC cells is particularly significant, given that this pathway is among the most frequently dysregulated in CRC—largely due to APC mutations—and represents a major driver of tumor progression, stemness, and metastasis (7, 34–36). Moreover, restoration of E-cadherin expression at both the mRNA and protein levels, along with reduced expression of *SLUG*, a core EMT transcription factor, points to enhanced cell adhesion and diminished invasive capacity (36, 37). Consistent with this phenotype, 1H5-treated SW620 cells also showed increased expression of CDH24, a cadherin linked to reinforced epithelial adhesion and reduced motility. These findings open new avenues for targeting metastatic CRC, where effective strategies to restrain Wnt/β-catenin activity remain a critical unmet need. The reduction in Wnt target-gene expression, despite unchanged nuclear β-catenin levels, may reflect downstream modulation of transcriptional output, including potential effects on TCF/LEF activity, cofactor interactions, or chromatin accessibility, rather than changes in β-catenin abundance (38). In parallel, we noted upregulation of DUSP5, a nuclear MAPK phosphatase that dampens ERK signaling; although SW620 is not an EGFR-driven cell line, elevated DUSP5 may reflect a compensatory response to broader MAPK pathway adjustments following ADAM10 inhibition.

In parallel, we evaluated 1H5 in different *in vivo* contexts. While robust therapeutic effects were observed in xenograft studies, treatment in an immunocompetent syngeneic model demonstrated reduced tumor growth, although the effect was not as pronounced as in human CRC xenografts, even though safety was confirmed through monitoring of body weight and MAHA responses. In line with this, we noted *in vitro* differences between KAP organoids carrying *Kras*^G12D^ and BAP organoids harboring *Braf*^V600E^, with the latter appearing more responsive. These findings suggest that underlying genetic context may influence therapeutic responsiveness. Together, these results underscore the importance of genetic background in shaping sensitivity to ADAM10 inhibition and warrant further investigation.

Collectively, our data demonstrate that conformation-specific targeting of ADAM10 holds strong therapeutic promise in CRC by suppressing ADAM10-dependent Notch and EGFR signaling. Importantly, and in contrast to earlier approaches using non-conformation-specific antibodies, our strategy did not result in detectable toxicities or unwanted side effects. This confers a high degree of specificity, minimizes off-target activity, and underscores the translational potential of 1H5 as a therapeutic candidate for CRC and other ADAM10-high malignancies. Beyond its activity as a blocking antibody, the selective binding of 1H5 to the active, tumor-associated conformation of ADAM10 also positions it as an attractive prototype for antibody–drug conjugate (ADC) development (37), where its unique target engagement could be leveraged to deliver cytotoxic payloads directly to malignant cells, thereby broadening its therapeutic utility.

## Materials and methods

### Cell culture and reagents

Human colorectal cancer cell lines COLO205, DLD-1, and SW620 (ATCC, USA) were maintained in RPMI-1640 medium supplemented with 10% fetal bovine serum (FBS) and 1% penicillin/streptomycin (P/S). Cells were cultured in a humidified incubator at 37°C with 5% CO_2_. FHC cells (ATCC CRL-1831), a non-transformed human fetal colon epithelial line originally established from normal fetal colon tissue, were cultured according to ATCC recommendations in DMEM/F12 medium supplemented with 10% FBS, 1% P/S, 10 ng/mL cholera toxin, 5 µg/mL insulin, 5 µg/mL transferrin, and 100 ng/mL hydrocortisone.

All cell lines were routinely tested and confirmed to be mycoplasma-free.

For 3D colorectal cancer (CRC) organoid cultures, KAP and BAP organoids, kindly provided by Dr. Daniel Dauch (University of Tübingen, Germany) (39), were maintained in Advanced DMEM/F12 medium supplemented with 20 mM L-glutamine, 10 mM HEPES, 1× penicillin/streptomycin (P/S), B27 supplement, and N2 supplement. The culture medium also contained N-acetylcysteine, mouse Noggin, and nicotinamide to support organoid growth. To enhance survival after passaging, the medium was supplemented with Y-27632, a ROCK inhibitor (HelloBio, UK), for two to three days. Organoids were cultured at 37°C in an incubator with 5% CO_2_.

The 1H5 IgG used throughout the study was expressed in Expi293 cells and purified from culture supernatants using Protein A–Sepharose followed by size-exclusion chromatography on an SD200 column (25).

Commercial ADAM10 antibodies used in this study included Abcam ab124695 (Abcam, USA), which is specific for human and mouse ADAM10 and was used for Western blotting. For flow cytometry, PE anti-human CD156 (BioLegend, USA) was used. Human IgG isotype control was purchased from Thermo Scientific (31154). Other commercial antibodies were purchased from Cell Signaling Technology (USA), including those against Notch1 (3608S), NICD1 (4147S), phospho-ERK (4370S), total ERK (4695S), phospho-EGFR (3777S), EGFR (4267S), E-cadherin (14472T), β-catenin (8480S), Lamin A/C (4777T), and GAPDH (2118S). Goat anti-rabbit IgG (H+L) HRP-conjugated secondary antibody (32460, Invitrogen, USA) and mouse IgG horseradish peroxidase–conjugated antibody (HAF007; R&D Systems, USA) were used as secondary antibodies.

### Alamar blue viability assay

Cell viability was assessed using the Alamar Blue assay (Biotium, #30025-1). Cells were seeded at 5,000 cells per well in 96-well flat-bottom plates and allowed to adhere overnight. The following day, cells were treated with increasing concentrations of 1H5 (5–40 µg/mL) or IgG control (40 µg/mL) in technical triplicates. After 48 and 72 h of treatment, 10% (v/v) Alamar Blue reagent was added directly to each well and incubated for 2–4 h at 37°C, protected from light. Fluorescence was measured using a microplate reader (excitation 560 nm, emission 590 nm). Background fluorescence from cell-free wells was subtracted, and viability was calculated by normalizing fluorescence values of treated wells to the corresponding IgG control. All experiments were performed in at least three independent biological replicates.

### Live cell imaging

Wild-type COLO205, DLD-1, and SW620 cells, along with shRenilla- and shADAM10-transduced COLO205, DLD-1, and SW620 cells (5 × 10³ cells per well), were seeded in 96-well plates and allowed to adhere overnight. Doxycycline (Sigma, USA) was added to the shRenilla and shADAM10 colorectal cancer cell lines to induce gene knockdown. Live-cell imaging was performed using the IncuCyte live-cell imaging system (Sartorius, Germany), capturing images every 4 h for a total of 96 h.

After 24 h of incubation, the anti-ADAM10 monoclonal antibody 1H5 was added at final concentrations of 5, 10, 20, and 40 μg/mL. Human IgG (40 μg/mL; Invitrogen, USA) was used as a negative control. The experiment was conducted in three independent replicates.

### Cell titer glo for viability assay in organoids

Organoids were seeded in 96-well plates at a density of 10–20 organoids per well (4,000–10,000 cells) in 25 μL of Matrigel. After allowing organoids to establish for 24 h, treatments were applied at final concentrations of 5, 10, 20, and 40 μg/mL. Following 48 and 72 h of treatment, organoid viability was assessed using the CellTiter-Glo^®^ 3D Cell Viability Assay (Promega) according to the manufacturer’s instructions. Briefly, an equal volume of CellTiter-Glo 3D reagent was added to each well, followed by mixing on an orbital shaker for 25 min at room temperature to ensure complete cell lysis and ATP release. Each assay included three independent biological replicates, with three technical replicate wells per treatment condition. Luminescence values were background-subtracted and normalized to IgG-treated controls.

### Western blotting

Cultured cells were lysed in RIPA buffer (G Biosciences, USA) containing 50 mM Tris-HCl (pH 7.4), 150 mM NaCl, 1% Triton X-100, 0.5% sodium deoxycholate, 0.1% SDS, 1 mM EDTA, and protease inhibitors. Protein concentration was determined using the BCA Protein Assay Kit (Thermo Fisher Scientific, USA).

For protein analysis, 20 µg of total protein lysate was subjected to SDS-PAGE using Mini-PROTEAN^®^ TGX™ Precast Protein Gels (4%–15%, Bio-Rad, USA), followed by transfer onto a PVDF membrane (iBlot™ Transfer Stack, Thermo Fisher Scientific, USA). Membranes were blocked with 5% milk in TBST and incubated with primary antibodies. After incubation with HRP-conjugated secondary antibodies, protein signals were detected using SuperSignal™ Femto Maximum Sensitivity Chemiluminescent Substrate (Thermo Fisher Scientific, USA).

### Flow cytometry

shRenilla- and shADAM10-transduced colorectal cancer cell lines were treated with doxycycline for 48 h to induce shRNA expression. GFP-positive populations were used as an indicator of effective knockdown. Following treatment, cells were detached using trypsin–EDTA (Gibco, USA), washed twice with ice-cold phosphate-buffered saline (PBS) containing 2% fetal bovine serum (FBS) (Gibco, USA), and resuspended in staining buffer. To assess surface ADAM10 expression, cells were incubated with PE-conjugated anti-human CD156 antibody at 4°C for 30 min in the dark. Flow cytometry was performed using the Attune™ NxT Flow Cytometer (Thermo Fisher Scientific, USA), and at least 10,000 events per sample were recorded. Data acquisition and compensation were performed using Attune NxT software (Thermo Fisher Scientific, USA). Further analysis was conducted using FlowJo™ v10 software (FlowJo LLC, USA), and the percentage of PE-positive cells was compared between shRenilla and shADAM10 groups.

### Construction of shRNA lentiviral vectors

To achieve inducible knockdown of ADAM10, shRNA sequences targeting the transcript were predicted using SplashRNA (http://splashrna.mskcc.org/). The knockdown efficiency of each shRNA was validated using a cell-based assay in which a GFP-expressing reporter cell line responsive to shRNAs was infected with single copies of retroviruses carrying miRE-shRNAs. GFP reduction was quantified using flow cytometry, and the shRNA demonstrating the highest knockdown efficiency was selected for further experiments.

The guide-strand sequences of the two selected shRNAs were as follows: shADAM10 #1 (TCTACTTTAAATTCATCACTGA); shADAM10 #2 (TTAAGTTAAAAATATCTGTTCA). The shRenilla control guide strand was TAGATAAGCATTATAATTCCTA. These sequences were cloned into the LT3GEPIR lentiviral vector, an all-in-one doxycycline-inducible system containing a Tet-On system with the rtTA3 transactivator and an shRNA expression cassette driven by a TRE3G promoter (26). The transactivator was constitutively expressed, while shRNA expression was induced upon doxycycline (Sigma, USA) administration at 1 mg/mL, allowing controlled knockdown of ADAM10.

Lentiviral particles were produced by co-transfecting HEK293T cells with the LT3GEPIR-shRNA plasmid, along with psPAX2 (packaging plasmid) and pMD2.G (envelope plasmid), using PEI (Polysciences, USA). Viral supernatants were collected 48 and 72 h post-transfection, filtered through a 0.45 µm filter, and used for transduction of COLO205, DLD-1, and SW620 colorectal cancer cell lines.

Cells were transduced with lentivirus in the presence of 8 µg/mL polybrene (Sigma, USA), and stable cell lines were selected using puromycin (Gibco, USA) at 1 µg/mL. Knockdown efficiency was confirmed by GFP expression, flow cytometry staining with PE-conjugated anti-human CD156 antibody, and Western blotting.

### Quantitative real-time polymerase chain reaction

Total RNA was extracted from cultured cells using TRIzol reagent (Life Technologies, USA) or the RNeasy Mini Kit (Qiagen, Cat. No. 74104) according to the manufacturer’s instructions. RNA was reverse-transcribed into complementary DNA (cDNA) using the QuantiTect Reverse Transcription Kit (Qiagen, Germany).

Quantitative real-time PCR (qRT-PCR) was performed using SYBR Green Master Mix (Thermo Fisher Scientific, USA) and gene-specific primers (listed in **Table 1**). Primer specificity was confirmed by melt-curve analysis and by verification of single amplicon products. Reactions were run on a QuantStudio™ real-time PCR system (Applied Biosystems, USA). Gene expression was analyzed using the ΔΔCt (comparative Ct) method, with GAPDH as the internal reference gene. Each reaction was performed in technical triplicate and repeated in at least three independent biological replicates.

**Table 1:** List of primers used for SYBR Green–based qRT-PCR analysis.

Primer name	Sequence
*HES1*	Forward primer: AACCAAAGACAGCATCTGAGCA
	Reverse primer: CCCAGCACACTTGGGTCTGT
*PYCR1*	Forward primer: TGCCTTGCATGTGCTGGAGAGT
	Reverse primer: GCTTCACCTTGTCCAGGATGGT
*SCD1*	Forward primer: CCTGGTTTCACTTGGAGCTGTG
	Reverse primer: TGTGGTGAAGTTGATGTGCCAGC
*LDHA*	Forward primer: GGATCTCCAACATGGCAGCCTT
	Reverse primer: AGACGGCTTTCTCCCTCTTGCT
*SLC7A5*	Forward primer: GCCACAGAAAGCCTGAGCTTGA
	Reverse primer: ATGGTGAAGCCGATGCCACACT
*MYC*	Forward primer: CCTGGTGCTCCATGAGGAGAC
	Reverse primer: CAGACTCTGACCTTTTGCCAGG
GAPDH	Forward primer: CTCTTGTGCTCTTGCTGGG
	Reverse primer: TAGGTAGGGGATCGGGACTC
S100A4	Forward primer: CTCAGCGCTTCTTCTTTC
	Reverse primer: GGGTCAGCAGCTCCTTTA
DKK1	Forward primer: GGTATTCCAGAAGAACCACCTTG
	Reverse primer: CTTGGACCAGAAGTGTCTAGCAC
NKD1	Forward primer: GAAGATGGAGAGAGTGAGCGAAC
	Reverse primer: GTCATACAGGGTGAAGGTCCAC
Vimentin	Forward primer: AGGCAAAGCAGGAGTCCACTGA
	Reverse primer: ATCTGGCGTTCCAGGGACTCAT
ZEB1	Forward primer: GGCATACACCTACTCAACTACGG
	Reverse primer: TGGGCGGTGTAGAATCAGAGTC
E-Cad	Forward primer: GCCTCCTGAAAAGAGAGTGGAAG
	Reverse primer: TGGCAGTGTCTCTCCAAATCCG
Slug	Forward primer: GGGGAGAAGCCTTTTTCTTG
	Reverse primer: TCCTCATGTTTGTGCAGGAG
NOTUM	Forward primer: CTCCATTTTACAAGCAGCAG
	Reverse primer: GCTCTTTCCTATCCTGTTCA
ASCL2	Forward primer: CGCCTACTCGTCGGACGACAG
	Reverse primer: GCCGCTCGCTCGGCTTCCG

### Transwell invasion assay

Cell invasion was assessed using Transwell chambers with 8 μm pore-size inserts (Corning Costar, Corning, NY, USA). Cells were suspended in serum-free medium and seeded into the upper chamber at a density of 25,000 cells per well. The lower chamber was filled with complete medium containing 10% fetal bovine serum (FBS) as a chemoattractant. After 24 h of incubation at 37°C with 5% CO_2_, non-invading cells on the upper surface of the membrane were carefully removed using a cotton swab.

Invaded cells on the lower membrane surface were fixed with 70% ethanol for 15 min at room temperature and then stained with DAPI (Abcam, USA) for nuclear visualization. Images were acquired using a fluorescence microscope (4× objective) from five randomly selected fields per insert.

For quantification, images were analyzed using ImageJ. The Analyze Particles function was applied after setting an appropriate threshold to accurately count invaded cells. The average cell count from five fields per insert was used for statistical analysis. The assay was performed in three independent biological replicates, each with triplicate inserts per condition.

### Wound healing assay

To evaluate the migratory capacity of metastatic colorectal cancer cells following treatment, a wound healing assay was performed using SW620 cells. Cells were seeded in 12-well plates and allowed to reach confluence over 24 h. A uniform scratch was then introduced into the cell monolayer using a sterile pipette tip, after which detached cells and debris were carefully removed by washing with phosphate-buffered saline (PBS). Cells were subsequently cultured under standard conditions for 24 and 48 h to allow migration into the wound area.

Images of the wounds were captured at 0, 24, and 48 h using an optical microscope at 4× magnification. Wound closure was quantified by measuring the migrated distance using ImageJ software (NIH, USA). Three independent biological replicates were performed, each with duplicate wells per condition. Wound area was normalized to the 0 h measurement.

### Animal studies

To establish xenograft models, 2 × 10^6^ DLD-1 cells per mouse were resuspended in 100 μL of phosphate-buffered saline (PBS; Corning, USA) and injected subcutaneously into the right flanks of 6-week-old female NU/J mice (Jackson Laboratory). For syngeneic models, 3 × 10^5^ KAP organoid cells per mouse were resuspended in 50% Matrigel (Corning, USA) and injected subcutaneously into 6-week-old female C57BL/6 mice. Each treatment group consisted of 4–5 mice. Tumor growth was monitored twice weekly for a total of seven injections, and xenograft volume was measured using calipers. Tumor volume was calculated using the formula V = ½ (length × width²). Once tumors reached 100 mm³, the anti-ADAM10 monoclonal antibody 1H5 was administered intraperitoneally, while human IgG was used as a control. Mouse body weight and general health were monitored daily.

At study endpoint or upon reaching humane endpoints, mice were humanely euthanized by gradual-fill CO_2_ inhalation in accordance with the American Veterinary Medical Association (AVMA) guidelines and SUNY Downstate IACUC requirements.

### MAHA ELISA assay

To assess the presence of mouse anti-human antibodies (MAHA), 25 μL of murine serum was collected from each mouse via submandibular bleed before and after 1H5 treatment at 10-day intervals. The MAHA ELISA assay (Eagle Biosciences, USA) was performed according to the manufacturer’s instructions. Absorbance was measured at 450 nm using a microplate reader. The concentration of anti-human IgG was quantified using a standard curve, and assay validity was confirmed using internal controls of known concentrations provided by the manufacturer.

### Bulk RNA sequencing and analysis

Total RNA was extracted using the Qiagen RNeasy Mini Kit according to the manufacturer’s protocol, including on-column DNase treatment. Libraries were generated by Novogene using poly(A) mRNA enrichment, followed by cDNA synthesis, end repair, adaptor ligation, and PCR amplification according to their standard workflow. Library quality was confirmed by Qubit quantification and Bioanalyzer fragment analysis. Sequencing was performed by Novogene on the Illumina NovaSeq X platform using paired-end 150 bp (PE150) chemistry. Raw reads were cleaned by removing adaptor sequences and low-quality bases, and sequencing quality was assessed with FastQC (v0.11.2). Clean reads were aligned to the human GRCh38.p13 reference genome using HISAT2 (v2.1.0), and aligned reads were assembled into transcripts with StringTie (v1.3.5). Raw gene-level counts were imported into DESeq2 (v1.28.1) for differential expression analysis. Genes with |log_2_ fold change| ≥ 1.5 and an adjusted p-value (padj) ≤ 0.05 were considered differentially expressed; non-coding genes and low-count transcripts were excluded from downstream analyses. All raw and processed RNA-seq data have been deposited in the NCBI Gene Expression Omnibus (GEO) under accession number GSE311253.

### Statistical analysis

Data are presented as mean ± standard deviation and analyzed using GraphPad Prism 10. For comparisons between two groups, the Mann–Whitney *U* test (non-parametric) was used, depending on data distribution. For comparisons among more than two groups with one variable, the Kruskal–Wallis test with Dunn’s *post hoc* test was used for non-parametric data. Two-way ANOVA was used when evaluating the effects of multiple variables, followed by Dunnett’s *post hoc* test. Statistical significance was defined as *p* < 0.05 unless otherwise indicated.

## Data Availability

All raw and processed RNA-seq data have been deposited in the NCBI Gene Expression Omnibus (GEO) under accession number GSE311253.
