# Supplementary material for: Inhibition of colorectal cancer progression through conformation-specific targeting of ADAM10 metalloprotease
Source: Front Oncol. 2026 Jan 27;15:1704436. doi: 10.3389/fonc.2025.1704436 (PMC12886026; doi:10.3389/fonc.2025.1704436)

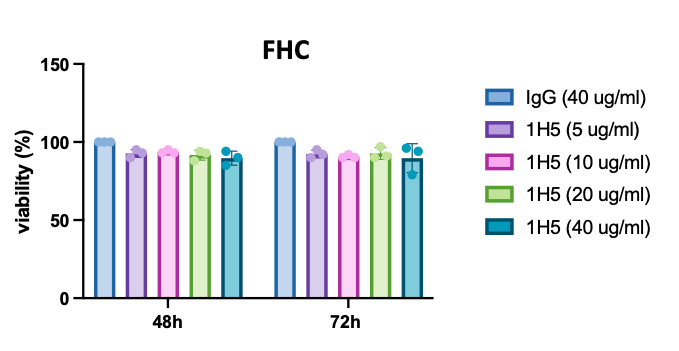
Supplementary Fig-1

**Supplementary Fig.1. Relative cell viability of FHC cells treated with increasing concentrations of 1H5 (5–40 µg/mL) for 48 and 72 hours. Cell viability was measured using a viability assay and normalized to IgG-treated controls. Data are shown as mean ± SD and analyzed using two-way ANOVA with Dunnett’s multiple-comparison test, with no statistically significant differences detected.**

Supplementary Fig.2


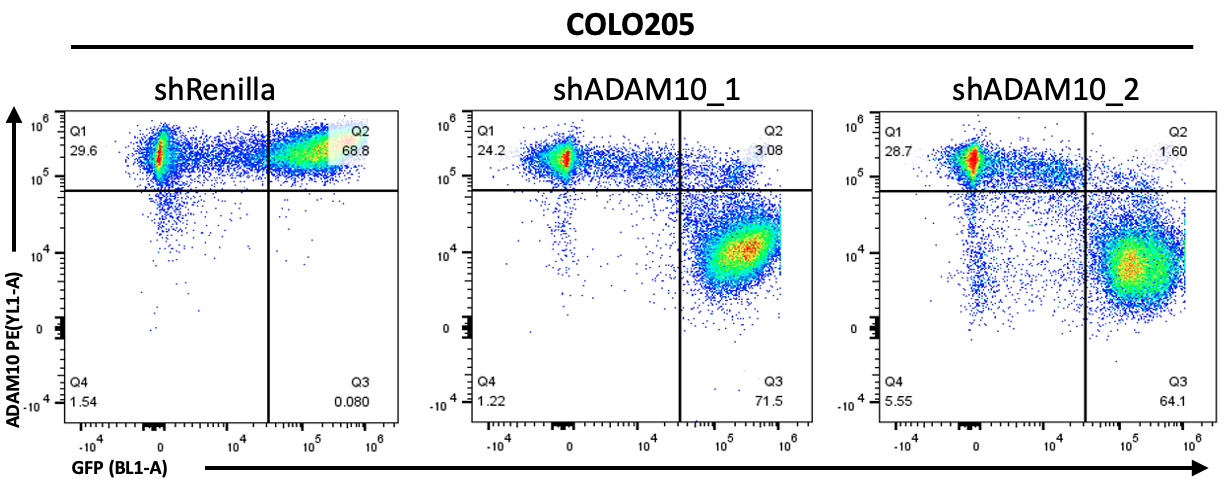

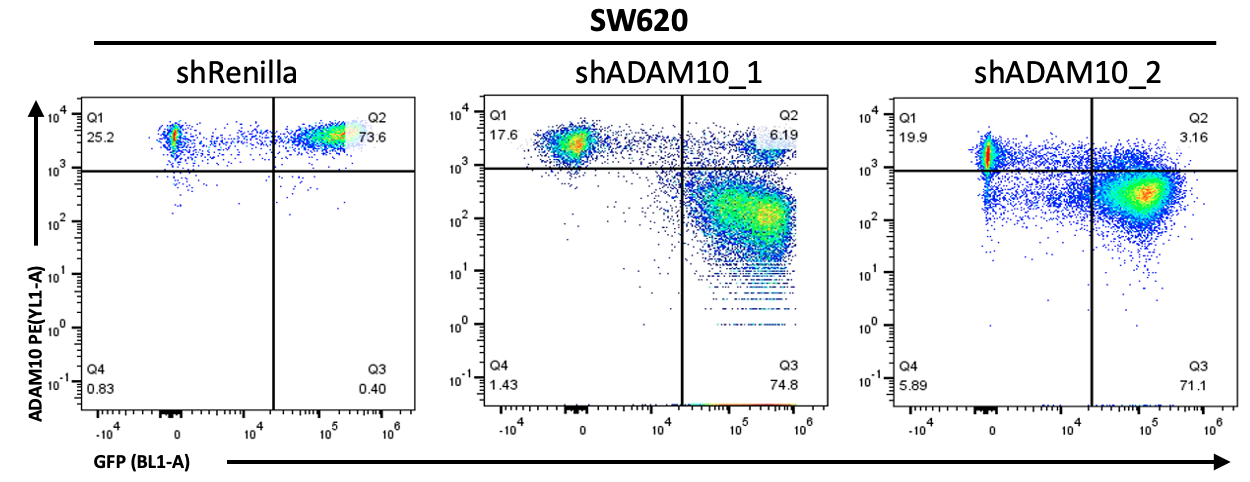

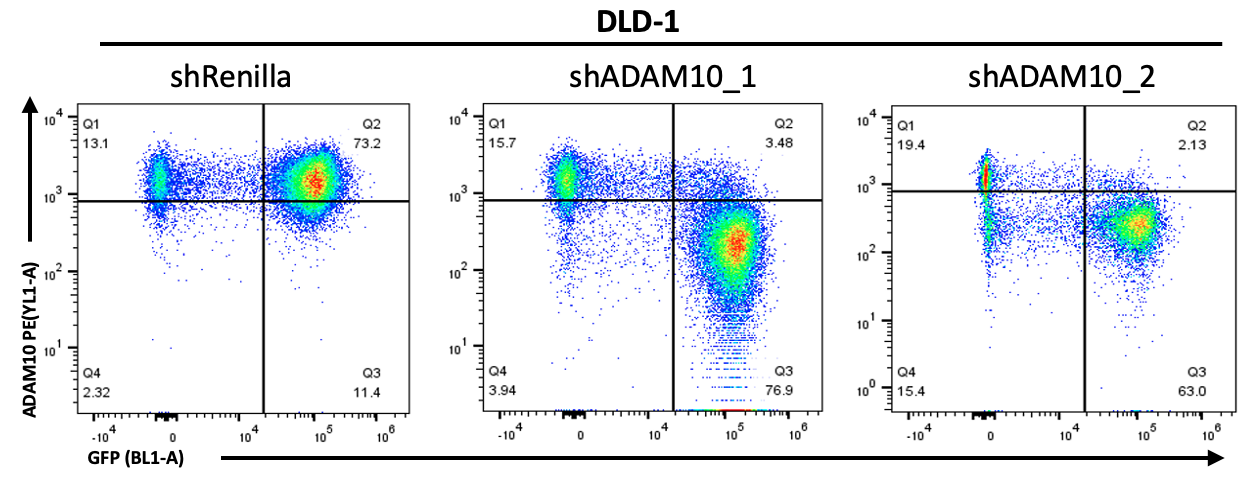


**GFP (BL1-A)**

**ADAM10 (YL1-A)**

**GFP (BL1-A)**

**ADAM10 (YL1-A)**

**GFP (BL1-A)**

**ADAM10 (YL1-A)**

**Supplementary Fig.2. Validation of doxycycline-inducible ADAM10 knockdown in colorectal cancer cell lines. (A) Flow cytometry analysis of COLO205, SW620, and DLD-1 cells transduced with doxycycline-inducible shRenilla control or two independent ADAM10-targeting shRNAs (shADAM10_1 and shADAM10_2). Cells were analyzed for GFP expression (indicating transduction) and surface ADAM10 levels using PE-conjugated anti-ADAM10 antibody.**

Supplementary Fig.3


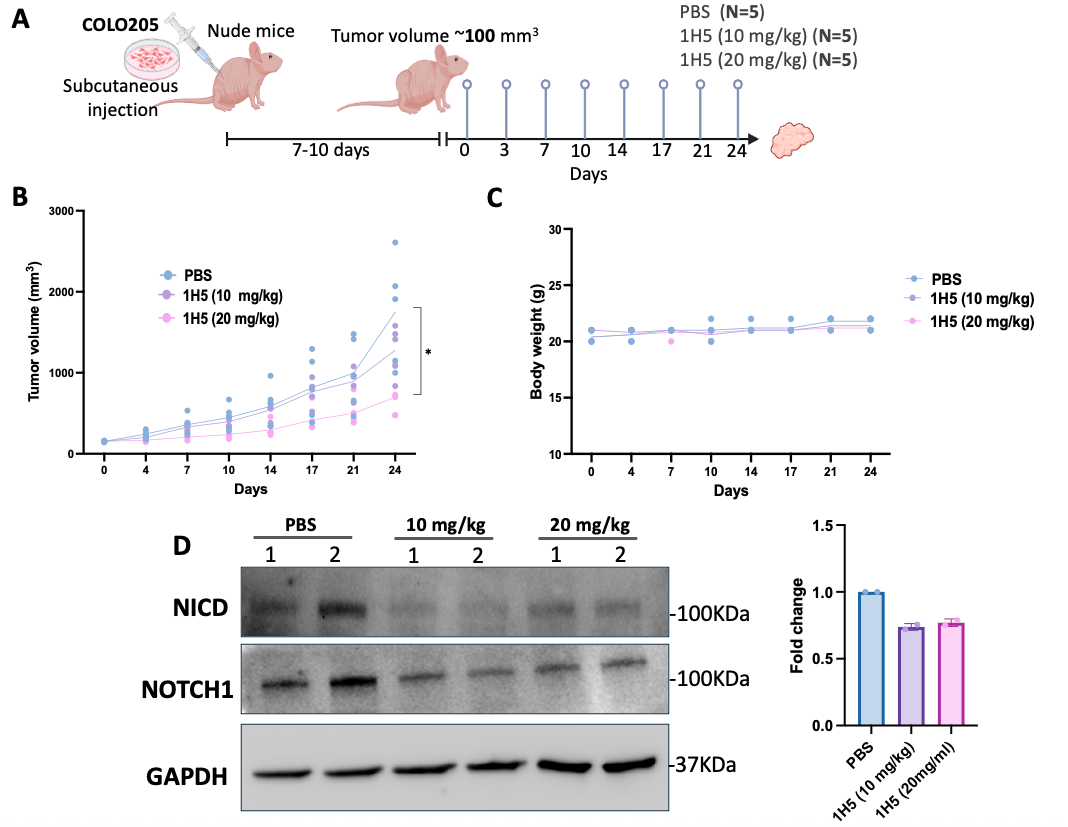


**Supplementary Fig. 3. *In vivo* evaluation of 1H5 in a subcutaneous COLO205 xenograft model of colorectal cancer.(A) Experimental timeline: Nude mice were subcutaneously injected with COLO205 cells and randomized into three groups (PBS, 1H5 at 10 mg/kg, and 1H5 at 20 mg/kg; n = 5 per group) once tumors reached approximately 100 mm³. Treatments were administered biweekly for 24 days.(B) Tumor volume measurements over the course of treatment. Final tumor volumes at day 24 were analyzed using the Kruskal–Wallis test with Dunn’s post-hoc comparisons.(C) Body weight monitoring throughout the study to assess potential treatment-related toxicity.(D) Western blot analysis of NICD and NOTCH1 in tumor lysates collected at the study endpoint. Western blotting was performed on two separate biological replicates per group. GAPDH was used as a loading control.**


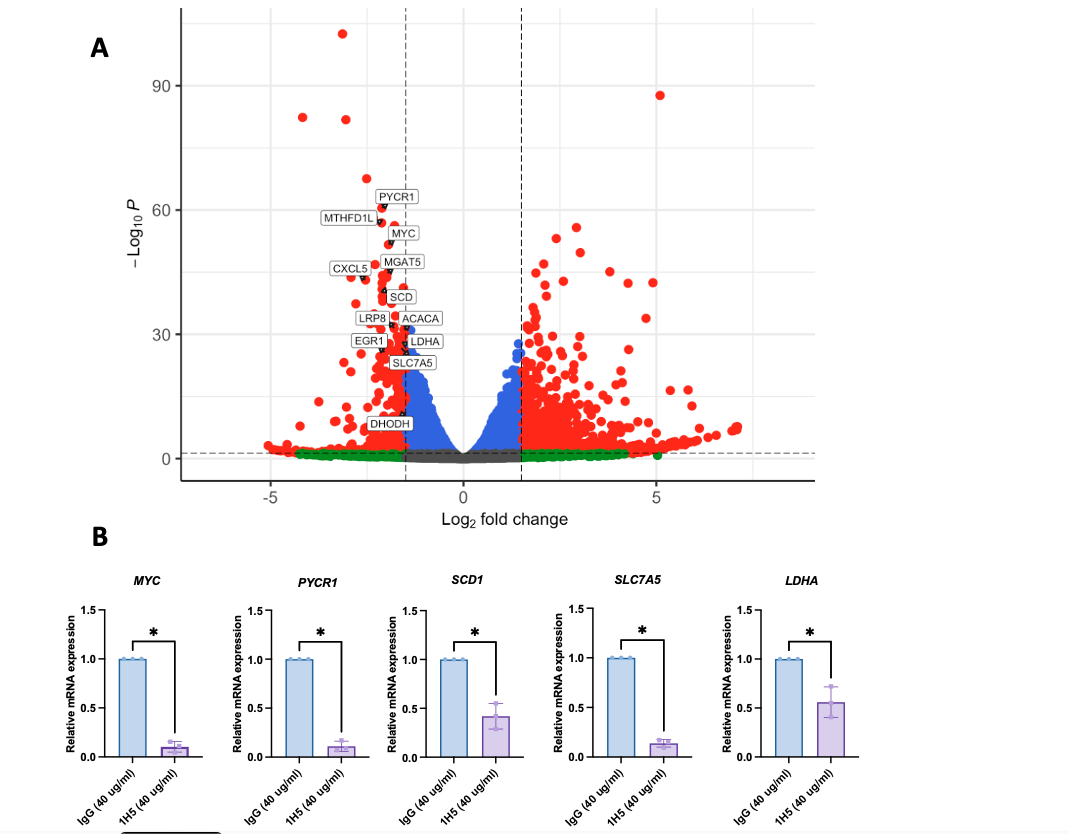
Supplementary Fig. 4

**Supplementary Fig. 4. Transcriptomic analysis and qRT-PCR validation of metabolic gene downregulation following 1H5 treatment in DLD-1 cells. (A) Volcano plot showing differentially expressed genes (DEGs) in DLD-1 cells treated with 40 µg/mL 1H5 for 48 hours compared with IgG control. DEGs were identified based on log₂ fold change ≥ ±1.0 and adjusted p-value ≤ 0.05. Key metabolic pathway genes are highlighted. (B) qRT-PCR validation of selected downregulated metabolic genes (MYC, PYCR1, SCD1, SLC7A5, and LDHA) in DLD-1 cells treated under the same conditions. Gene expression was normalized to a housekeeping gene and presented relative to IgG-treated controls. Data are presented as mean ± SD. Statistical significance was assessed using the Kruskal–Wallis test followed by Dunn’s multiple-comparison test; *p < 0.05.***

Supplementary Fig.5


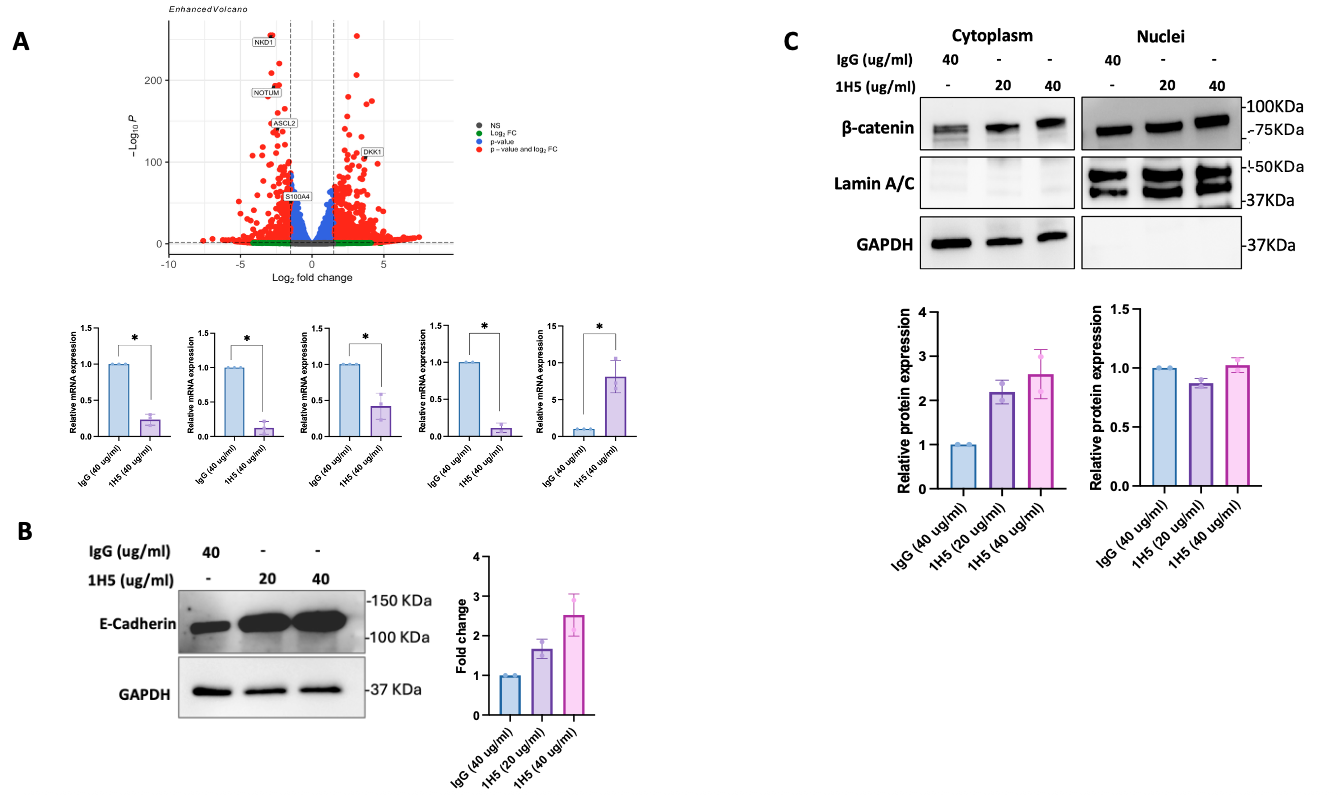


**Supplementary Fig. 5. 1H5 regulates E-cadherin expression and β-catenin localization in SW620 cells. (A) KEGG pathway enrichment analysis showing significantly downregulated (blue) and upregulated (red) pathways in SW620 cells treated with 1H5 (40 µg/mL) compared with IgG control. Dot size represents the number of genes per pathway, and the x-axis indicates adjusted p-values. Gene expression validation of selected pathway genes was performed by qRT-PCR using three biological replicates, shown as mean ± SD and analyzed using the Kruskal–Wallis test; *p < 0.05. (B) Western blot analysis of E-cadherin levels in SW620 cells treated with 1H5 (20 or 40 µg/mL) or IgG control (40 µg/mL) for 48 hours. Western blotting was performed on two biological replicates. GAPDH served as a loading control. (C) Subcellular fractionation and Western blot analysis of β-catenin in cytoplasmic and nuclear fractions of SW620 cells following treatment with 1H5 (20 or 40 µg/mL) or IgG control (40 µg/mL). Lamin A/C and GAPDH were used as nuclear and cytoplasmic markers, respectively. Quantification of β-catenin in each fraction is shown below. Western blot analyses were performed on two biological replicates.**

Supplementary Fig.6


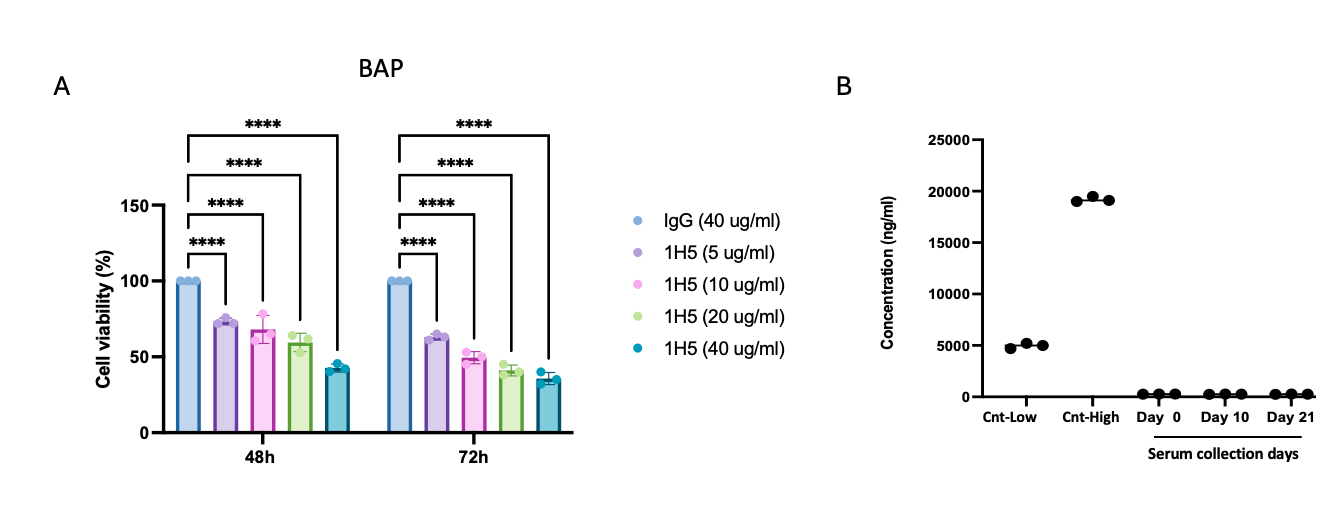


**Supplementary Fig. 6. Cell viability of BAP organoids and MAHA ELISA analysis. (A) Cell viability of BAP organoids treated with 1H5. BAP organoids were treated with IgG control (40 µg/mL) or increasing concentrations of 1H5 (5–40 µg/mL) for 48 h and 72 h. Viability was measured using the CellTiter-Glo 3D assay and normalized to the IgG control. Data represent mean ± SD from three independent biological replicates, each with technical triplicates. Statistical analysis was performed using two-way ANOVA followed by Dunnett’s multiple comparisons test. (B) Mouse anti-human antibody (MAHA) ELISA assay. Serum samples collected from 1H5-treated mice at the indicated timepoints were analyzed for MAHA levels using an indirect ELISA. Plates were coated with human IgG, and bound mouse antibodies were detected using HRP-conjugated anti-mouse IgG. Absorbance at 450 nm was converted to concentration using a standard curve. Each point represents an individual mouse.**

**Table 1.** List of primers used for SYBR Green–based qRT-PCR analysis.

| **Primer name** | **Sequence** |
| --- | --- |
| *HES1* | Forward primer: AACCAAAGACAGCATCTGAGCA |
|  | Reverse primer: CCCAGCACACTTGGGTCTGT |
| *PYCR1* | Forward primer: TGCCTTGCATGTGCTGGAGAGT |
|  | Reverse primer: GCTTCACCTTGTCCAGGATGGT |
| *SCD1* | Forward primer: CCTGGTTTCACTTGGAGCTGTG |
|  | Reverse primer: TGTGGTGAAGTTGATGTGCCAGC |
| *LDHA* | Forward primer: GGATCTCCAACATGGCAGCCTT |
|  | Reverse primer: AGACGGCTTTCTCCCTCTTGCT |
| *SLC7A5* | Forward primer: GCCACAGAAAGCCTGAGCTTGA |
|  | Reverse primer: ATGGTGAAGCCGATGCCACACT |
| *MYC* | Forward primer: CCTGGTGCTCCATGAGGAGAC |
|  | Reverse primer: CAGACTCTGACCTTTTGCCAGG |
| GAPDH | Forward primer: CTCTTGTGCTCTTGCTGGG |
|  | Reverse primer: TAGGTAGGGGATCGGGACTC |
| S100A4 | Forward primer: CTCAGCGCTTCTTCTTTC |
|  | Reverse primer: GGGTCAGCAGCTCCTTTA |
| DKK1 | Forward primer: GGTATTCCAGAAGAACCACCTTG |
|  | Reverse primer: CTTGGACCAGAAGTGTCTAGCAC |
| NKD1 | Forward primer: GAAGATGGAGAGAGTGAGCGAAC |
|  | Reverse primer: GTCATACAGGGTGAAGGTCCAC |
| Vimentin | Forward primer: AGGCAAAGCAGGAGTCCACTGA |
|  | Reverse primer: ATCTGGCGTTCCAGGGACTCAT |
| ZEB1 | Forward primer: GGCATACACCTACTCAACTACGG |
|  | Reverse primer: TGGGCGGTGTAGAATCAGAGTC |
| E_Cad | Forward primer: GCCTCCTGAAAAGAGAGTGGAAG |
|  | Reverse primer: TGGCAGTGTCTCTCCAAATCCG |
| Slug | Forward primer: GGGGAGAAGCCTTTTTCTTG |
|  | Reverse primer: TCCTCATGTTTGTGCAGGAG |
| NOTUM | Forward primer: CTCCATTTTACAAGCAGCAG |
|  | Reverse primer: GCTCTTTCCTATCCTGTTCA |
| ASCL2 | Forward primer: CGCCTACTCGTCGGACGACAG |
|  | Reverse primer: GCCGCTCGCTCGGCTTCCG |

**Western blot raw data**

**
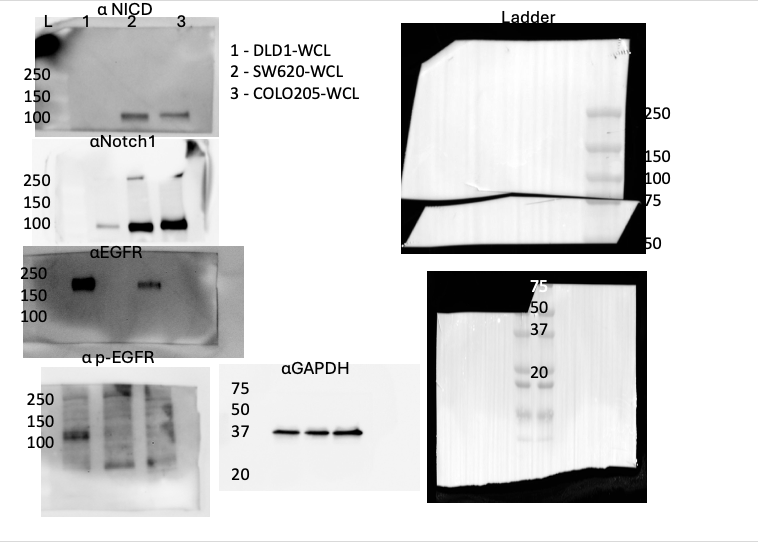
Figure 3-A**

**
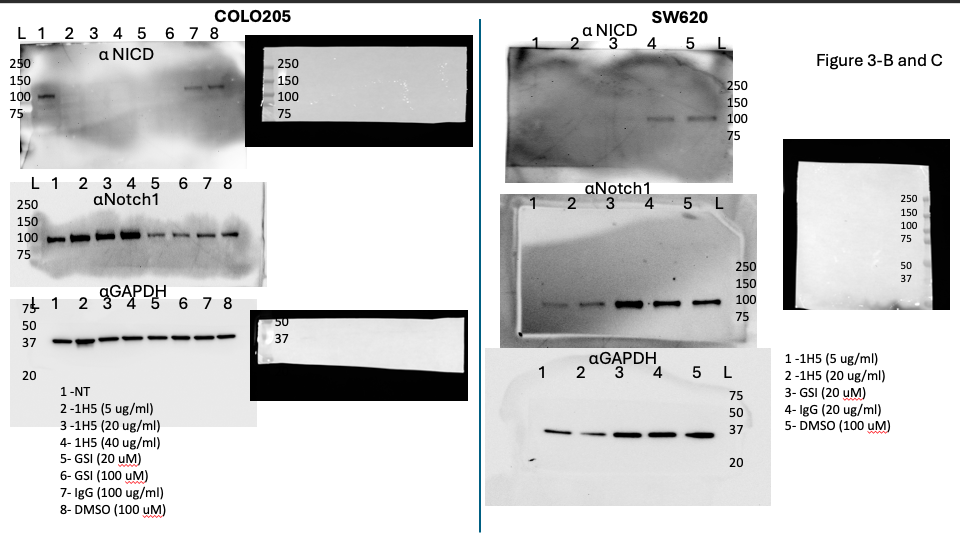
**

**
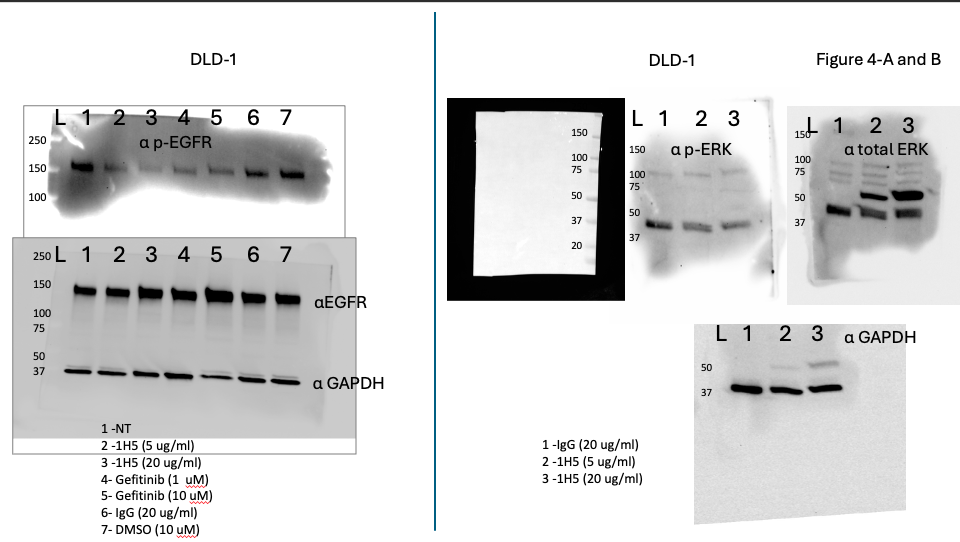
**

**
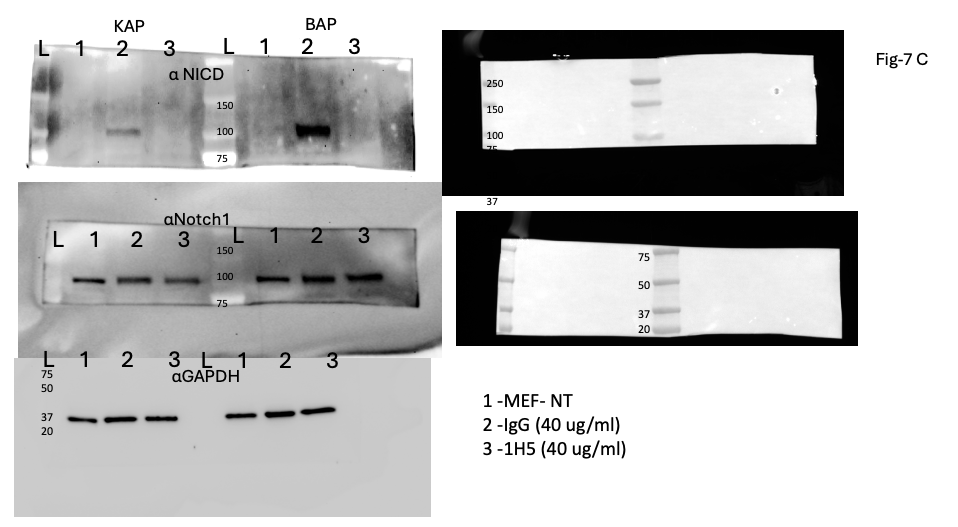
**


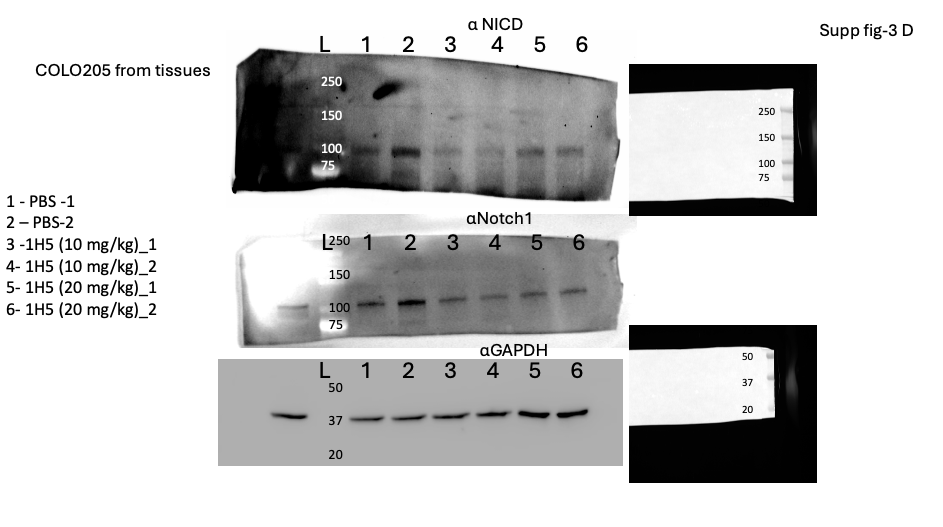


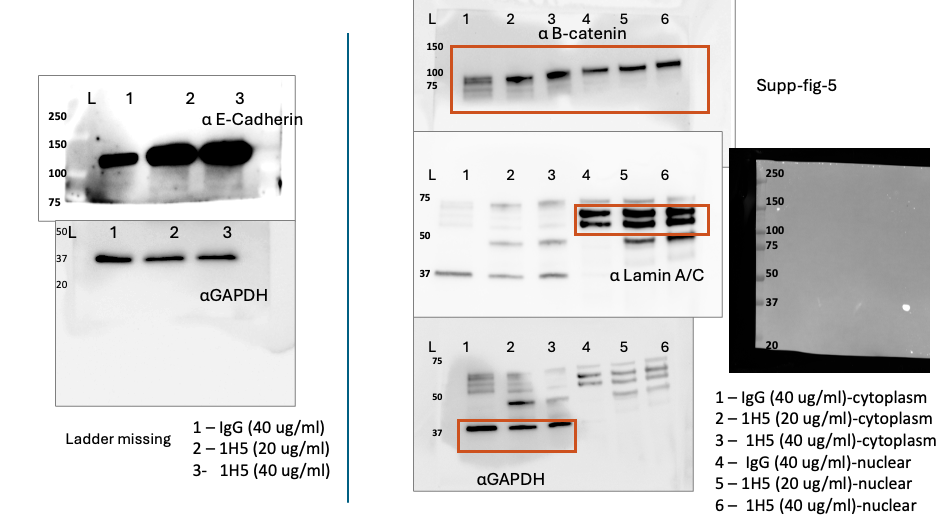

Supplement: Supplementary file 1 [file DataSheet1.docx]
